# Development and computational analysis of high dimensional spectral flow cytometry data for the resolution of innate lymphoid cells in the mammary tumor microenvironment

**DOI:** 10.3389/fimmu.2026.1730567

**Published:** 2026-01-27

**Authors:** Hobin Seo, Jingna Xue, Qiutong Huang, Megan Kinzel, Amisha Verma, Ngan Huynh, Zahra Jamila Ikra, Douglas J. Mahoney, Jongbok Lee, Sorana Morrissy, Nicolas Jacquelot

**Affiliations:** 1 Riddell Centre for Cancer Immunotherapy, Arnie Charbonneau Cancer Institute, Arthur J.E. Child Comprehensive Cancer Centre, University of Calgary, Calgary, AB, Canada; 2 Department of Biochemistry & Molecular Biology, Cumming School of Medicine, University of Calgary, Calgary, AB, Canada; 3 Department of Microbiology, Immunology & Infectious Diseases, Cumming School of Medicine, University of Calgary, Calgary, AB, Canada; 4 Alberta Children’s Hospital Research Institute, Calgary, AB, Canada; 5 Calvin, Phoebe, and Joan Snyder Institute for Chronic Diseases, University of Calgary, Calgary, AB, Canada

**Keywords:** adaptive lymphocytes, bioinformatics, breast cancer, innate immunity, innate lymphoid cells, spectral flow cytometry, T cells, tumor immunology

## Abstract

Spectral flow cytometry has ushered in a new era in immunology. Through the improvement of the resolution of surface and intracellular protein expression, this approach enables in depth characterization of rare immune cell subsets, such as innate lymphoid cells (ILCs), in health and disease. Due to their heterogeneity, the identification of ILCs requires the use of many lineage marker antibodies for non-ILC exclusion, together with the analysis of several transcription factor expression profiles for ILC subset distinction. Such intricacies toward their identification and their scarcity in tissues have been key factors directly limiting their characterization, particularly during tumor development and progression. We developed, optimized and validated a 25-parameter spectral flow cytometric panel for the identification of mouse ILC subsets and characterization of their phenotype and proliferation capabilities in mouse mammary tumors. The use of conjugated antibodies coupled to different fluorochromes for the analysis of lineage marker expression further allows the identification and characterization of γδ T cells, CD4^+^ and CD8^+^ αβ T cells, as well as CD19^+^ B cells. Furthermore, we built a bioinformatics pipeline for unbiased immune cell clustering and marker expression analysis. We assessed this panel and downstream bioinformatics analyses on two spectral flow cytometers and found no difference in immune cell identification and clustering save for slight variations in marker intensity, inherent to the specificities of the instrument. These findings highlight the robustness of our developed approach for the identification of innate lymphoid cells in tumors, a method that can be easily implemented for day-to-day analysis of ILCs and other rare immune cell subsets.

## Introduction

The interrogation of population diversity within tissues using single-cell approaches continues to revolutionize our understanding of complex biological processes, heterogeneity in cell behaviors within previously thought homogeneous populations, and system interactions in health and disease. Aside from single-cell ATACseq and RNAseq to investigate genome accessibility and gene expression, respectively, flow cytometry is a widely used approach for single-cell analysis of protein expression, utilizing monoclonal antibodies coupled to fluorescent tags for cell marker detection and quantification (1). While conventional flow cytometry still represents a fast, reliable, and relatively low-cost approach, spectral flow cytometry is now a commonly used technique for capturing the complex heterogeneity within mixed populations (2). The recent advent of spectral flow cytometry allows for a better resolution of fluorophores with similar emission spectra but distinct off-peak emissions by collecting the full emission spectrum unique to each fluorophore (2). This advancement in resolving spectrally similar fluorophores, referred to as unmixing, allows for the analysis of up to 50 markers simultaneously (3). This represents a significant opportunity for the deep characterization and profiling of cells within the immune system, and for the identification of distinct tissue- or disease-dependent phenotypes and cell functions (3–6). However, the establishment of such complex antibody panels remains challenging and requires an optimized combination of antibodies and fluorophores to accurately resolve all markers, particularly those with low antigen expression. Furthermore, there is a need for the development of bioinformatics pipelines to process and analyze high-dimensional spectral flow data, functioning as a plug-and-play approach that can be easily fine-tuned to specific datasets and needs.

The innate lymphoid cell (ILC) family is composed of a diverse spectrum of immune cells that lack the expression of antigen-specific receptors (7). They play diverse roles in tissue homeostasis, host defense, tissue repair and remodelling, and inflammation (8). Despite increased understanding of their function in health and disease, how they shape tumor development and progression and response to treatments remains unclear (9, 10). The complex nature of the tumor microenvironment and the paucity of ILCs infiltrating tumors make the study of ILC function in cancer challenging. Together, these features complicate the analysis of these immune cells in cancer, calling for the development and optimization of robust spectral flow panels for single-cell analysis of tumor-infiltrating ILC subsets.

The ILC family is divided into five subsets – natural killer (NK) cells, type 1, 2 and 3 ILCs (ILC1s, ILC2s, ILC3s) and lymphoid tissue inducer cells (LTis) – characterized by distinctive developmental pathways, transcription factor and effector molecule expression and biological activities (7). ILCs lack lineage marker expression, but it is pertinent that flow cytometric antibody panels incorporate these markers to identify T cell, B cell and myeloid cell populations and exclude them from downstream ILC analyses. Unlike NK cells and ILC1s, which express surface markers NKp46 and NK1.1, ILC2s and ILC3s are identified by their expression of transcription factors GATA3 and RORγt. Within each subset, considerable diversity in phenotype and effector function has been revealed through single-cell transcriptomic and proteomic analyses, adding another level of complexity (11–16). Depending on the tissue of interest, ILCs may exhibit differences in surface marker expression. Lung ILC2s, for instance, express ST2, the receptor for interleukin (IL)-33, but not IL-17RB, the receptor for IL-25. In contrast, intestinal ILC2s express IL-17RB but not ST2 (14, 16, 17). To investigate ILC behavior and functional status across different tissues in health and disease, there is a need for complex flow cytometric panels with the ability to detect a variety of surface molecules and transcription factors.

**Figure 1:**
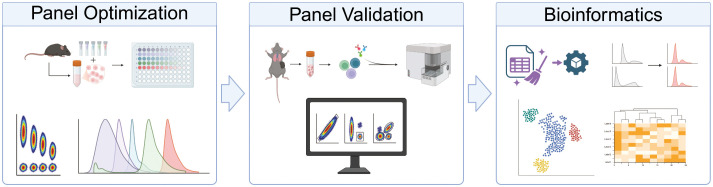
Workflow overview. Panel optimization. Selected antibodies were titrated on single-cell suspensions of murine tissues to optimize marker resolution. Panel validation. The staining panel was tested on lung single-cell suspensions and implemented to identify ILCs in mammary tumor tissues. Bioinformatics. Flow cytometric data were subject to clean-up and quality control steps for computational high-dimensionality reduction and clustering. Graphical abstract generated in BioRender.

Here we report the design, optimization, and testing of a 25-parameter, 24-color spectral flow cytometry panel to accurately define ILC subsets in tumors and other murine tissues, and the implementation of an automated bioinformatics pipeline for the analysis of tissue-resident leukocytes (Live CD45^+^) (**Figure 1**). The analysis of tumor-infiltrating innate and adaptive lymphocytes is presented here using the mammary-specific polyomavirus middle T antigen overexpression mouse model (MMTV-PyMT, hereinafter referred to as PyMT), a mammary cancer model in which developing mammary lesions closely recapitulate the progression of human disease (18–20). After unbiased clustering, we identified 13 lymphoid cell subsets, including NK cells, ILC1s, ILC2s, and ILC3s, and various sub-populations. In addition, we tested this staining panel on two spectral flow cytometers with no significant changes in immune cell frequency and numbers, highlighting the robustness of our established panel and bioinformatics approach.

## Materials and methods

### Mice

C57BL/6J mice were purchased from Jackson Laboratory (JAX stock #000664). *PyMT* (*MMTV-PyMT*) mice were obtained from Professor Pamela Ohashi, University of Health Network. *PyMT* mice were originally developed by Professor William Muller, McGill University (18). Mice were maintained on a C57BL/6J background by crossing C57BL/6J female mice (Jackson Laboratory, JAX strain #000664) to PyMT transgenic male mice. Mice were bred and maintained under specific pathogen-free conditions at the University of Calgary, under a 12 h:12 h light:dark cycle at 20–24°C and 30–50% humidity. Female mice expressing the *PyMT* transgene with confirmed mammary tumors were analyzed. Experiments were approved by the University of Calgary Health Sciences Animal Care Committee (under protocol AC23-0003, AC23-0054, and AC23-0162) and performed in accordance with the guidelines of the Canadian Council on Animal Care.

### Cell culture and tumor inoculation

Various tissues and tumor models were used for antibody titration and validation. The MC38 colorectal cancer and MCA205 fibrosarcoma cell lines were maintained at 37°C and 5% CO_2_ in complete media consisting of Roswell Park Memorial Institute (RPMI) 1640 (Gibco) containing 10% heat-inactivated FCS (Gibco), 1mM sodium pyruvate (Gibco) 2 mM L-Glutamine (Gibco), 50 mM β-mercaptoethanol (Gibco), 100 U/mL penicillin, and 100 mg/mL streptomycin (Gibco). Cells tested negative for *Mycoplasma*. C57BL/6J mice were shaved and 8 x 10^5^ MC38 or MCA205 cells resuspended in sterile 1X PBS (Gibco) were inoculated subcutaneously on the flank. Tumor size was measured routinely using calipers every two to three days and humane endpoint was reached when tumors were 15 mm in any direction or showed ulceration.

### Tissue processing

Mice were euthanized with CO_2_ or via cervical dislocation and spleens, small intestines, lungs and tumors were collected into plates containing ice-cold 1X PBS. Tissues were kept on ice until processing. *Lungs* were minced using scissors in 5 mL RPMI 1640 media containing 100 µg/mL DNAse I and 1 mg/mL Collagenase IV (Worthington Biochemicals). Tissues were digested under gentle agitation at 37°C for 45 minutes. After vortexing samples, digested tissues were filtered using a 70 μm cell strainer, washed with 10 mL 1X PBS, and centrifugated at 1500 rpm for 5 minutes. Cell pellets were resuspended in 1 mL ACK buffer for red blood cell lysis. After 2–3 minutes incubation at room temperature, suspensions were topped up with 9 mL 1X PBS and cells were centrifugated at 1500 rpm for 5 minutes. After discarding the supernatant, cells were resuspended in 600 μL of 1X PBS and kept on ice until antibody staining. *Tumors* harvested from *PyMT* mice at 14–16 weeks of age were first weighed, then minced, digested, and filtered as per lungs above. Cell pellets were resuspended in 1X PBS at a final concentration of 250 mg tumor per mL and kept on ice until antibody staining. *Spleens* were homogenized and filtered using a 70 μm cell strainer, washed with 10 mL 1X PBS and centrifugated at 1500 rpm for 5 minutes. Following red blood cell lysis with 1 mL ACK buffer for 2–3 minutes as per lungs above, cell pellets were resuspended in 5 mL 1X PBS. *Small intestines* were cleared of Peyer’s patches and fat tissue was removed. Following the removal of fecal content, tissues were washed with 1X PBS and minced to 2 mm pieces. Minced tissues were washed again with 1X PBS and dissociated in 20 mL Hank’s Balanced Salt Solution (HBSS) media (Gibco) containing 2% vol/vol FBS and 5 mM EDTA under gentle agitation at 37°C for 45 minutes. After vortexing samples, dissociated tissues were filtered using a 70 μm cell strainer and washed with 1X PBS. Tissues were digested in 8 mL in RPMI containing 2% vol/vol FBS, 100 μg/mL DNAse I, 1 mg/mL Collagenase IV, and 0.2 U/mL Dispase (Worthington Biochemicals) under gentle agitation at 37°C for 45 minutes. After vortexing samples, digested tissues were filtered using a 70 μm cell strainer, washed with 1X PBS, and centrifugated at 1700 rpm for 7 minutes. Cell pellets were resuspended in 6 mL 40% Percoll (Cytiva) and 4 mL 80% Percoll was underlaid. Cells were centrifugated at 2200 rpm for 20 minutes at low acceleration and no brake. Cell fraction at the interface was collected and washed with 1X PBS, and centrifugated at 1700 rpm for 10 minutes. Cell pellet was resuspended in 200 µL 1x PBS and kept on ice until antibody staining.

### Antibody staining panel

To select the appropriate antibody-fluorescent conjugate pairs, we characterized each marker as either primary, secondary, or tertiary (**Table 1**). We paired markers to fluorescent conjugates (**Table 2**) with the following in consideration. First, we paired tertiary markers preferentially to bright conjugates, with consideration for new commercially available conjugates with very distinct spectral signatures. For instance, R718 in place of AF700. R718 has lower secondary peak emission than AF700, which improves unmixing quality. Furthermore, the emission maxima of fluorochromes conjugated to tertiary markers should not overlap with the natural autofluorescent signature of tissue type. We found that lymphocytes in the lung and tumor autofluoresce with peak emission in the same channels as BUV496 and BV510 (**Supplementary Figure 1A**). CD11b and CD90.2 have high expression profiles that are not impacted by tissue autofluorescence and were paired to these conjugates, respectively. In addition, the signal from certain conjugates can either spread into another conjugate or impact its staining index. The spillover spreading matrix (SSM) and stain index reduction (SIR) can be evaluated to ensure that the resolution of tertiary markers is preserved. For example, GATA3 staining on BV711 is negatively impacted by RB705, whereas GATA3 on PE-Cy7 shows improved resolution (**Supplementary Figure 1B**). When assigning conjugates for the remaining primary and secondary markers, it is important to pair co-expressed markers with conjugates that are spectrally dissimilar to each other. For instance, CD3 and CD4 are co-expressed by CD4^+^T cells and if put on overlapping conjugates, the resulting combination can significantly impact staining resolution (**Supplementary Figure 1C**). Finally, careful consideration should be taken regarding the use of conjugates with secondary emission peaks in channels across multiple lasers, as they significantly induce spread and reduce the resolution of affected parameters. We have incorporated RB705 in this panel, which has the same peak emission as PerCP-eF710, but significantly less background.

**Table 1:** Antibody classification based on expression profile.

Classification	Criteria	Markers
Primary	Markers with distinct and high expression, often binary (“on” or “off”) and classify major cell lineages	CD3, CD11b, CD45, CD8a, CD4, NKp46, TCRβ, CD19, NK1.1
Secondary	Markers with moderate to high expression density, often exhibit gradient expression and classify broad subsets of cells	CD49a, Ki67, CD25, CD90.2, KLRG1, CD117, EOMES, CD127,
Tertiary	Rare markers of interest, markers with dim and/or variable expression	IL-17RB, RORγt, ST2, PD-1, 4-1BB, LAG-3, GATA3

**Table 2:** Antibody characteristics.

Specificity	Fluorochrome	Clone	Vendor	RRID	Application	Cytek titration	Cytek µL antibody/test	Sony titration	Sony µL antibody/test
CD3e	BUV395	145-2C11	BD Biosciences	AB_2738278	Surface	1:200	0.25	1:100	0.5
CD11b	BUV496	M1/70	Thermo Fisher Scientific	AB_2925322	Surface	1:400	0.125	1:100	0.5
IL-17RB	BUV563	6B7	BD Biosciences	AB_2873042	Surface	1:100	0.5	1:100	0.5
CD49a	BUV615	Ha31/8	BD Biosciences	AB_2875123	Surface	1:400	0.125	1:800	0.0625
Ki67	BUV737	SolA15	Thermo Fisher Scientific	AB_2896016	Intracellular	1:200	0.25	1:100	0.5
CD25	BUV805	PC61.5	Thermo Fisher Scientific	AB_2896102	Surface	1:800	0.0625	1:200	0.25
RORγt	BV421	Q31-378	BD Biosciences	AB_2687545	Intracellular	1:100	0.5	1:100	0.5
CD90.2	BV510	53-2.1	BioLegend	AB_2561395	Surface	1:400	0.125	1:800	0.0625
IL-33R (ST2)	BV605	U29-93	BD Biosciences	AB_2742841	Surface	1:100	0.5	1:100	0.5
CD45	BV786	30-F11	Thermo Fisher Scientific	AB_2925726	Surface	1:800	0.0625	1:800	0.0625
CD8a	FITC	53-6.7	BD Biosciences	AB_394569	Surface	1:800	0.0625	1:1000	0.05
KLRG1	AF532	2F1	Thermo Fisher Scientific	AB_2815282	Surface	1:200	0.25	1:200	0.25
CD117	RB705	2B8	BD Biosciences	AB_3685851	Surface	1:400	0.125	1:800	0.0625
CD4	RB744	GK1.5	BD Biosciences	AB_3685781	Surface	1:800	0.0625	1:1000	0.05
CD279 (PD-1)	RB780	RMP1-30	BD Biosciences	AB_3688159	Surface	1:100	0.5	1:100	0.5
CD335 (NKp46)	PE	29A1.4	BD Biosciences	AB_1727466	Surface	1:200	0.25	1:200	0.25
EOMES	PE-CF594	X4-83	BD Biosciences	AB_2916484	Intracellular	1:400	0.125	1:800	0.0625
CD127	PE-Fire640	S18006K	BioLegend	AB_2927992	Surface	1:800	0.0625	1:800	0.0625
GATA3	PE-Cy7	L50-823	BD Biosciences	AB_1645544	Intracellular	1:100	0.5	1:100	0.5
CD137 (4-1BB)	APC	17B5	Thermo Fisher Scientific	AB_2573162	Surface	1:100	0.5	1:100	0.5
NK1.1	SparkNIR685	S17016D	BioLegend	AB_2910321	Surface	1:400	0.125	1:1000	0.05
CD223 (LAG-3)	R718	C9B7W	BD Biosciences	AB_2917150	Surface	1:100	0.5	1:100	0.5
Viability	Zombie NIR	–	BioLegend	–	Surface	1:1000	0.05	1:1000	0.05
TCRβ	APC-eF780	H57-597	Thermo Fisher Scientific	AB_1272173	Surface	1:800	0.0625	1:400	0.125
CD19	APC-eF780	eBio1D3 (1D3)	Thermo Fisher Scientific	AB_10853189	Surface	1:1000	0.05	1:800	0.0625

### Flow cytometry

*Viability staining*. In a 96-well conical (V) bottom plate, single-cell suspensions were incubated in 50 μL 1X PBS with reconstituted Zombie NIR dye (BioLegend) at a 1:1000 dilution for 30 minutes on ice. Cells were washed with 1X PBS 2% vol/vol FBS (henceforth called flow cytometry buffer), centrifugated at 1700 rpm for 3 minutes and supernatant was discarded. *Fc receptor blocking*. Cells were resuspended in 50 μL flow cytometry buffer with anti-mouse CD16/CD32 (clone 93, eBioscience) at a final concentration of 2.5 μg/mL and incubated for 20 minutes on ice. Cells were then centrifugated at 1700 rpm for 3 minutes and supernatant was discarded. *Surface antibody staining*. Cells were resuspended in 50 μL flow cytometry buffer containing surface antibodies (**Table 2**) and incubated for 30 minutes on ice. Cells were washed with flow cytometry buffer, centrifugated at 1700 rpm for 3 minutes and supernatant was discarded. *Cell fixation and permeabilization*. Cells were resuspended in 50 μL Foxp3 Transcription Factor Fixation/Permeabilization solution (eBioscience) at a 1:3 concentrate to diluent ratio, as per manufacturer’s protocol, and incubated for 30 minutes on ice. Cells were washed with either flow cytometry buffer or 1X Permeabilization Buffer (eBioscience), centrifugated at 1700 rpm for 3 minutes and supernatant was discarded. *Intracellular antibody staining*. Cells were resuspended in 50 μL 1X Permeabilization Buffer containing intracellular antibodies (**Table 2**) and incubated for 45 minutes on ice. Cells were washed with flow cytometry buffer and supernatant was discarded. *Sample acquisition and analysis*. Stained samples were resuspended in 200 μL flow cytometry buffer and acquired on a 5-laser Cytek Aurora System (Cytek Biosciences) or 6-laser Sony ID7000 Spectral Cell Analyzer (Sony Biotechnology). For direct comparison between the two instruments, we divided samples into two for staining and acquisition. Single color reference controls were prepared on UltraComp eBeads™ Plus Compensation Beads (Thermo Fisher Scientific) and BD™ SpectraComp™ Unmixing and Compensation Particles (BD Biosciences), or a 1:1 live to heat killed single-cell suspension for Zombie NIR. For cell count enumeration, CountBright™ Absolute Counting Beads (Thermo Fisher Scientific) were added to samples prior to acquisition. Samples were spectrally unmixed using single color reference controls and the autofluorescent signatures extracted from unstained controls as parameters. Unmixed samples were analyzed with FlowJo software v10.10.0.

### Bioinformatics analysis

*Data cleaning and import into R.* Sample data were cleaned up on FlowJo and live CD45^+^ populations were exported to FCS3.0 format. The FCS3.0 files were loaded into R using *FlowCore (*21), *FlowViz (*22), and *FlowVS (*23) packages as a collection of flowFrame files. Formatting data using *flowSet* allows efficient bulk processing and a neat R file environment, especially when there is a significant number of files. Importantly, only FCS files with the same parameter configuration can be imported into the same *flowSet*. *Transformation and Quality control.* Arcsinh transformations were performed using the FlowVS (23) package, and co-factors of 2000 were used for the lung samples, 4000 and 5000 were used for the tumor samples run on Cytek and Sony, respectively. Appropriate cofactors need to be tested, and different cofactors can be applied to different fluorescence channels. Using optimal co-factors, data transformation stabilizes the variance within each fluorescence channel, homogenizing cell populations for analyzing downstream biologically relevant clusters. Quality control was then performed using the *PeacoQC (*24) package. *Normalization of batch effects.* To normalize fluorescence intensities across technical replicates, we used the *CytoNorm (*25) package and performed batch correction by using aggregated files from multiple samples and tissue types. *Clustering and dimensionality reduction.* Adapted from the approach described in Spasic et al. (26), we incorporated the *Seurat (*27) packages for the clustering of flow cytometric datasets. CSV files were exported from pre-processed FCS files using the *Phenoflow (*28) package before being imported as Seurat Objects using *Seurat*. After scaling of the data and principal component analysis (PCA), we used lineage markers as *features* to calculate neighbors and define clusters. Next, we performed dimensionality reduction using uniform manifold approximation (UMAP) to better preserve the data structure, which maintains the spatial relationship between cells and clusters. *Data Integration.* We used the *Harmony (*29) method for data integration. The R scripts and associated FCS files were deposited onto *https://github.com/JacquelotLab* for easy implementation and reproducibility of the analysis.

### Statistical analyses

Data analyses and representations were performed either with R software or Prism (GraphPad version 10.1.0). Statistical analyses were two-sided. Unpaired comparisons between two groups were performed using Mann-Whitney tests. Paired comparisons between two groups were performed using Wilcoxon tests. Results are shown as the mean ± SD. *p*-values were two-sided with 95% confidence intervals and considered significant at *p* < 0.05.

## Results

### Designing and validation of a 25-parameter spectral flow cytometric panel for the identification of innate lymphoid cells

**Figure 2:**
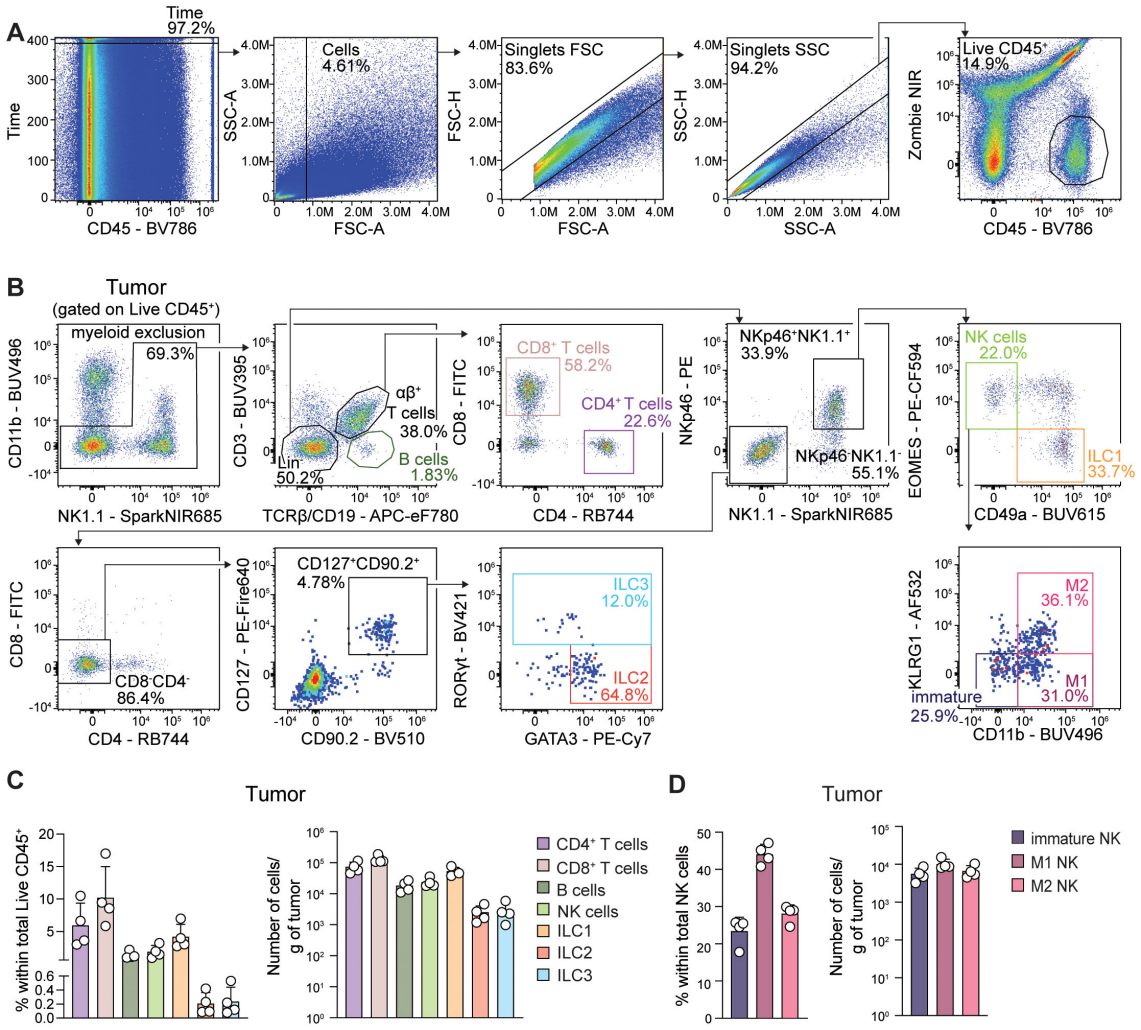
All ILC subsets infiltrate PyMT mammary tumors. Flow cytometric analyses of PyMT mammary tumor samples. **(A)** Representative cleanup of unmixed tumor sample data to identify live CD45^+^ cells. **(B)** Representative gating strategy used to identify the major lymphocyte subsets in tumors. NK cells were identified as CD3^-^TCRβ^-^CD19^-^NK1.1^+^NKp46^+^EOMES^+^CD49a^-^; immature NK cell subset was identified as KLRG1^-^CD11b^-^NK cells; M1 NK cell subset was identified as KLRG1^-^CD11b^+^NK cells; M2 NK cell subset was identified as KLRG1^+^CD11b^+^NK cells; ILC1s were identified as CD3^-^TCRβ^-^CD19^-^NK1.1^+^NKp46^+^CD49a^+^EOMES^-^; ILC2s were identified as CD3^-^TCRβ^-^CD19^-^NK1.1^-^NKp46^-^CD4^-^CD8^-^CD90.2^+^CD127^+^GATA3^+^RORγt^-^; ILC3s were identified as CD3^-^TCRβ^-^CD19^-^NK1.1^-^NKp46^-^CD4^-^CD8^-^CD90.2^+^CD127^+^RORγt^+^. **(C)** Proportion of each cell subset as a percentage of total live CD45^+^ leukocytes (left) and cell count per gram of tumor (right). **(D)** Proportion of NK cell subsets as a percentage of total NK cells (left) and cell count per gram of tumor (right). Bar graphs show the mean ± SD of 4 biological replicates. Data show one experiment out of two performed (N = 3–4 biological replicates/experiment).

The identification of ILCs relies on the absence of surface lineage markers and the use of lineage-defining transcription factors. As such, our panel incorporates markers to characterize a broad array of lymphocytes in addition to ILCs. In contrast to most previously published spectral flow cytometric ILC panels where lineage markers are grouped into one color for quick negative selection (6, 30, 31), our lineage cocktail consists of lineage-defining antibodies conjugated to spectrally distinct fluorochromes (**Table 2**). Using a 5-laser Cytek Aurora, the 25 commercially available antibodies we selected on 24 colors (**Table 2**) were first titrated on single-cell suspensions of murine tissues to determine staining resolution and antibody concentration for optimal staining (**Supplementary Figure 2**). We then tested this antibody staining panel on lung samples (**Supplementary Figure 3**) and were able to identify CD4^+^ and CD8^+^ T cells (CD3^+^TCRβ^+^), and B cells (CD19^+^). We assigned TCRβ and CD19 the same fluorophore due to their mutually exclusive expression profiles, reducing the complexity of the panel. By plotting against CD3, we were able to accurately identify both CD19^+^ B cells and CD3^+^TCRβ^+^ T cells. We used NK1.1 and NKp46, both expressed by group 1 ILCs, and stained for EOMES and CD49a to separate the population into NK cells and ILC1s, respectively. After ensuring the remaining lymphocytes do not express other lineage markers, CD90.2- and CD127-expressing cells were further characterized as GATA3^+^RORγt^-^ ILC2s, or RORγt^+^ ILC3s. We implemented this panel to identify ILCs in mammary tumor tissue (**Figure 2**) isolated from PyMT mice.

### Increased ILC1 but decreased ILC2 frequencies in PyMT mammary tumors compared to lungs

To identify the various ILC subsets in mammary tumor samples, we implemented a gating strategy onto the unmixed sample data (**Figure 2**). Briefly, after irregularities in flow rate, debris, and doublets were excluded, live CD45^+^ leukocytes were identified (**Figure 2A**). Using our validated manual gating strategy, we identified group 1 ILCs in tumors as CD3^-^TCRβ^-^CD19^-^NK1.1^+^NKp46^+^, further subdivided into NK cells or ILC1s based on EOMES and CD49a expression, respectively. In both lung and tumor samples, CD11b and KLRG1 expression was assessed for the detection of immature (CD11b^-^KLRG1^-^) and mature M1 (CD11b^lo^KLRG1^-^) and M2 (CD11b^lo^KLRG1^+^) NK cell subsets (32). Then, ILC2s were characterized as CD3^-^TCRβ^-^CD19^-^NK1.1^-^NKp46^-^CD4^-^CD8^-^CD90.2^+^CD127^+^GATA3^+^RORγt^-^ while ILC3s were identified as CD3^-^TCRβ^-^CD19^-^NK1.1^-^NKp46^-^CD4^-^CD8^-^CD90.2^+^CD127^+^RORγt^+^ (**Figure 2B**; **Supplementary Figure 3B**). In the lung, amongst the four ILC subsets, NK cells, particularly the M2 subset, and ILC2s constituted the major populations (**Supplementary Figure 3C**), while in mammary tumors, NK cells, largely of the M1 phenotype, and ILC1s represented most of the ILC infiltrate (**Figures 2C, D**).

### PyMT-infiltrating ILC2s show increased PD-1 expression and enhanced proliferative capacity

**Figure 3:**
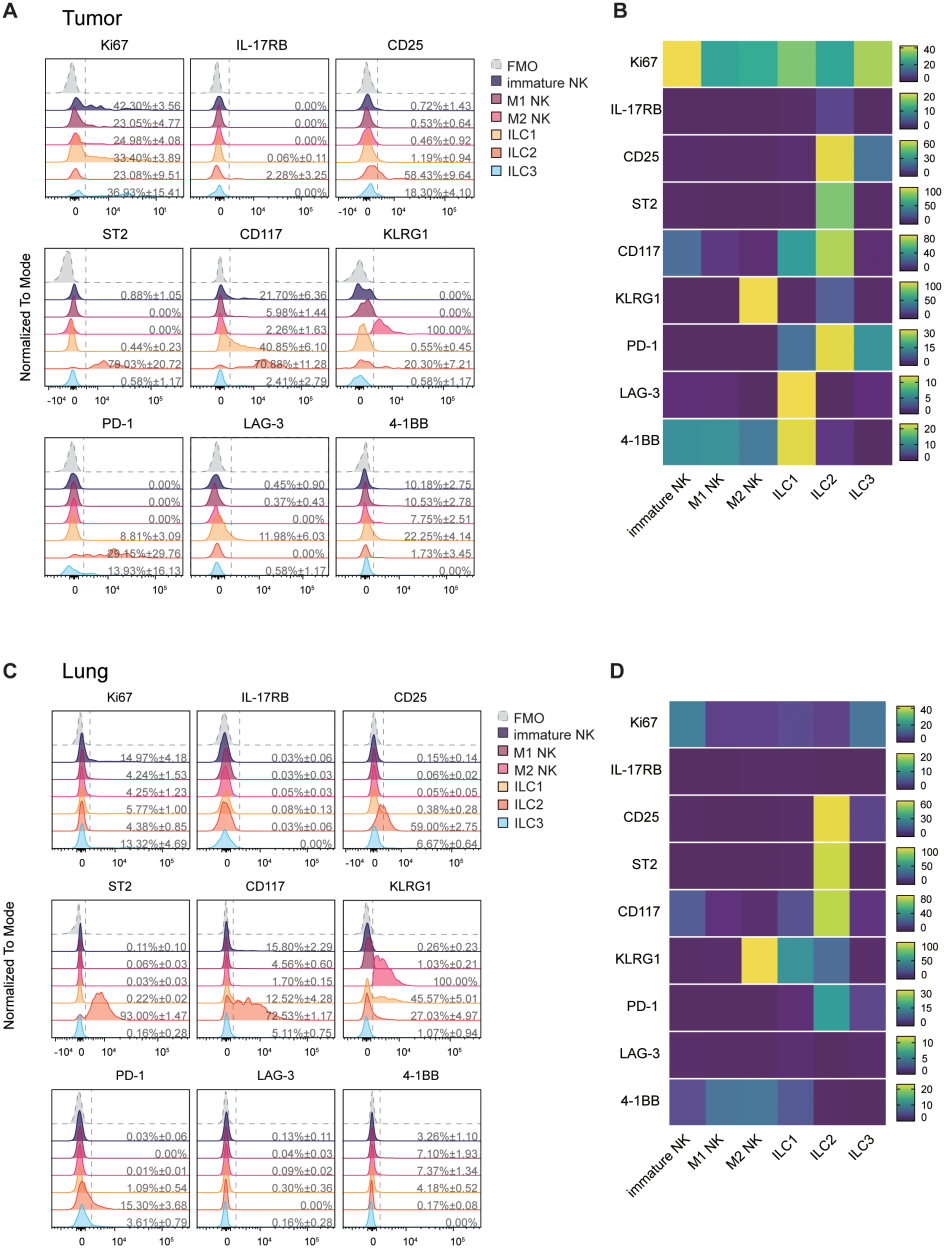
Mammary tumor-infiltrating ILC2s express high levels of PD-1. **(A)** Representative flow cytometric histograms showing individual marker expression on tumor-infiltrating ILC subsets. Mean expression ± SD are indicated. **(B)** Heatmap representing mean surface and intracellular marker percent positive expression in tumor ILC subsets from 4 biological replicates. **(C, D)** Same as A-B showing lung ILC subsets. Data show one experiment out of two performed (N = 3–4 biological replicates/experiment).

It is now widely recognized that ILCs are heterogenous populations. Depending on signals they receive from their environment, ILCs may exhibit significant plasticity in their phenotype and surface marker expression. As such, we incorporated markers to unravel the diversity within these populations across different tissue types. We observed that immature NK cells express higher levels of Ki67 compared to the other ILC subsets, and proliferation capacity in tumors was markedly higher in all ILCs compared to those in the lung (**Figure 3**). CD25 expression was mostly restricted to ILC2s in both tissues, although there was a minor population of CD25^+^ ILC3s (**Figure 3**). Unsurprisingly, ST2 expression was exclusive to ILC2s (**Figure 3**). Approximately 70% of tumor and lung ILC2s expressed CD117, and we observed a higher proportion of CD117^+^ immature NK cells and CD117^+^ ILC1s in the tumor compared to their lung counterparts (**Figure 3**). Interestingly, KLRG1 was expressed by nearly half of lung ILC1s, whereas KLRG1^+^ ILC1s were absent in the tumor (**Figure 3**). 4-1BB expression was largely exclusive to group 1 ILCs, and LAG-3 was only found to be expressed on a small number of tumor ILC1s, but not NK cells infiltrating mammary PyMT tumors, as previously reported by our group (33) (**Figure 3**). We observed increased PD-1^+^ ILC2s in mammary tumors, similar to our previous observations in melanoma (34), although a smaller subset of ILC2s in the lung also expressed PD-1 (**Figure 3**). The increase of PD-1 expression on tumor-infiltrating ILC2s mirrored that seen in T cells (**Supplementary Figure 4**), the latter of which has been well described in the context of anti-tumor immunity and immunotherapies (35). Amongst all ILC subsets, ILC2s have the highest levels of PD-1 in mammary tumors (**Figure 3**).

### Unbiased high-dimensional analysis revealed heterogeneity in immune cell populations

**Figure 4:**
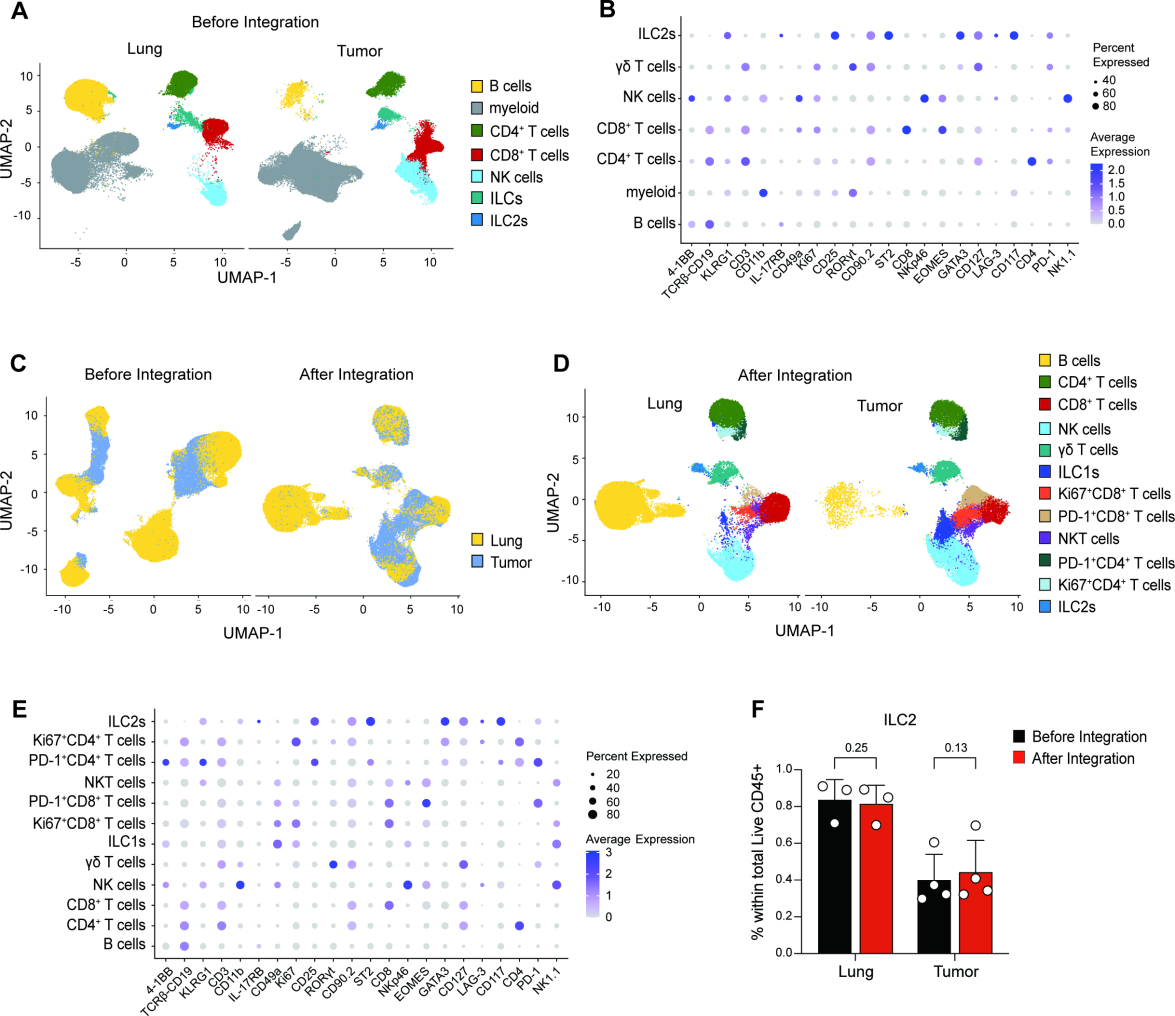
Identification of rare and unique lymphoid cell subsets in lungs and mammary tumor samples. Computational bioinformatics analyses of lung and mammary tumor samples isolated from PyMT mice. Uniform Manifold Approximation and Projection (UMAP) plots showing labelled cell populations in the lung and tumor before **(A, C)** and after integration **(C, D)**. Clustering using 159,751 cells. **(B, E)** Dotplots showing the expression level of indicated proteins in each cell cluster before **(B)** and after integration **(E)**. The size of the dots represents the proportion of cells that express indicated markers and the color of the dots denotes the average marker expression with blue showing the highest expression intensity. **(F)** Bar graph showing the percentage of ILC2s out of live CD45^+^ cells in the lung and tumor, before and after integration. Data show mean ± SD of one experiment out of two performed (N = 3–4 biological replicates/experiment). Wilcoxon matched-pairs signed rank test. Exact *p* values shown.

Analysis of flow cytometric data by gating strategy is a reliable way to identify proportions of ILCs and their expression profiles, all the while preserving the ability to characterize the phenotypes of adaptive lymphocytes (**Supplementary Figure 4**). However, the increased number of parameters that can be accurately unmixed by spectral flow cytometry allows for the analysis and visualization of data using similar bioinformatics approaches as the ones used to analyze high dimensional single-cell datasets. To that aim, we implemented an unbiased bioinformatics pipeline for high-dimensional flow cytometric analysis. We first cleaned and exported live CD45^+^ cell data from FlowJo, removing autofluorescence parameters and the upstream channels used to gate on live CD45^+^ cells (forward and side scatter, Zombie NIR, CD45), and saved them as individual new FCS files. The data were imported into R, transformed, quality controlled, normalized, and scaled (**Supplementary Figure 5**). Unsupervised clustering was conducted, and cells were visualized with UMAP (**Figure 4A**). Clusters were merged and labeled based on their protein profiles visualized by dotplots (**Figure 4B**), heatmap (**Supplementary Figure 6**) and feature plots (**Supplementary Figures 7**-**8**). Using marker expression for immune cell labelling, this initial clustering identified myeloid cells, B cells, CD4^+^ and CD8^+^ T cells, NK cells, ILC2s, and other ILC subsets. Because of technical variation in staining among different tissue types, the same cell types showed spatial differences between tumor and lung. Therefore, we used an integration method to eliminate variation inherent to differences in autofluorescence and staining intensities between tissues (**Figure 4C**). Additionally, we removed the CD11b^+^ myeloid population to improve the efficiency and accuracy of lymphoid cell re-clustering, since the panel lacked sufficient myeloid-related markers. After integration, spatial variation between tissues was resolved. With the exclusion of myeloid populations, we were able to identify additional cell subtypes (**Figures 4C, D**; **Supplementary Figures 9**-**10**), including ILC1s, γδ T cells, and Ki67^+^ and PD1^+^ αβ CD4^+^ and CD8^+^ T cell subsets. After quantification of the proportion of each cell type, we found that the percentage of ILC2s remained similar before and after integration (**Figures 4E, F**), indicating that the integration process did not affect clustering accuracy but, instead, improved data resolution with the identification of additional unique subsets.

### Robustness of our workflow for ILC identification between spectral flow cytometers

**Figure 5:**
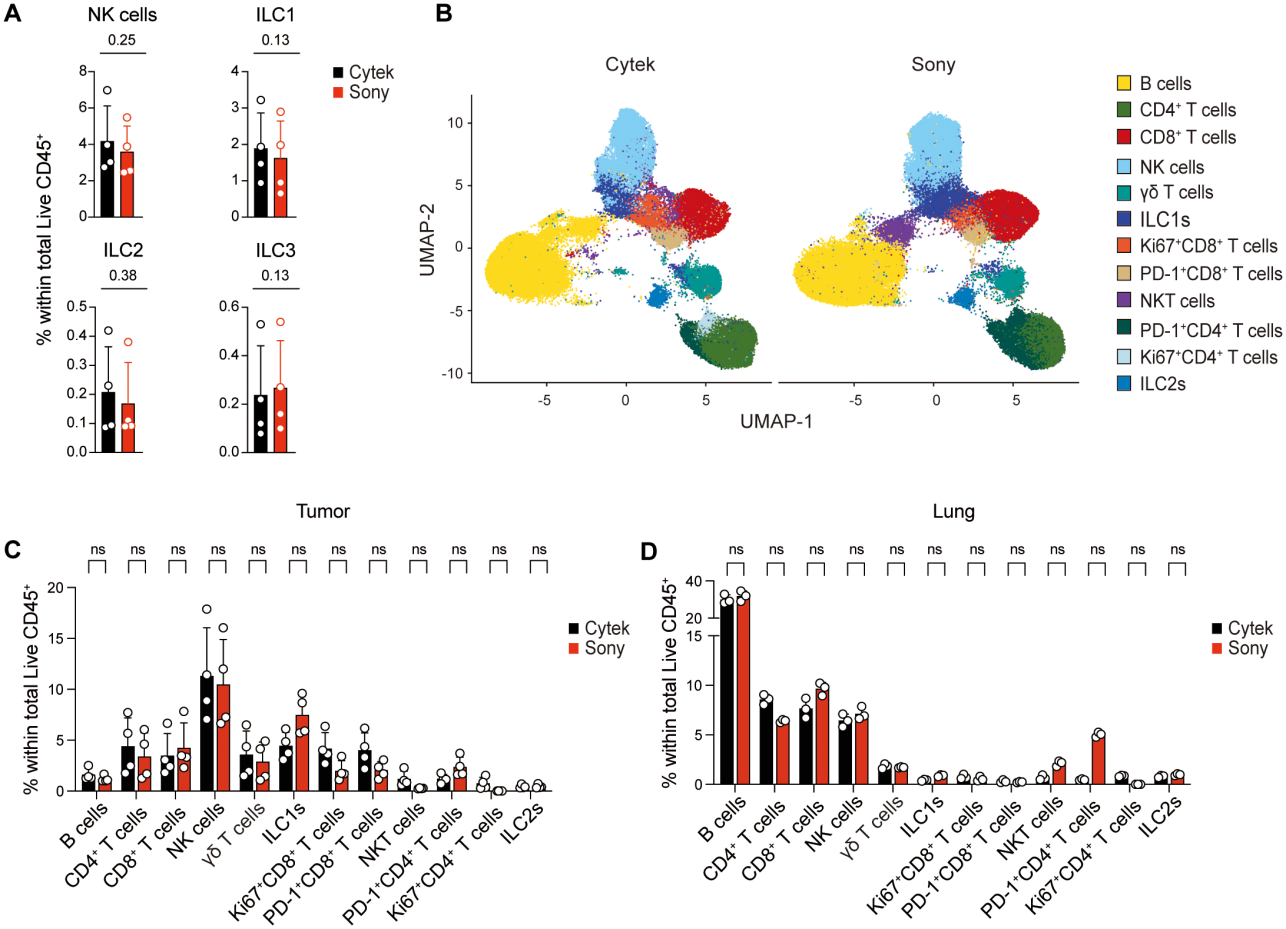
ILC identification across flow cytometric platforms. **(A)** Comparison of tumor-infiltrating lymphocyte frequencies as a percentage of total CD45^+^ leukocytes between data acquired on the Cytek Aurora and Sony ID7000 flow cytometers. **(B)** UMAP plots of lung and tumor cell populations after integration from data acquired on the Cytek Aurora (left) and the Sony ID7000 (right). Comparisons of cell subset proportions between Cytek and Sony platforms within lung **(C)** and tumor **(D)** data, quantified by computational analysis. Data show mean ± SD of one experiment out of two performed (N = 3–4 biological replicates/experiment). Wilcoxon matched-pairs signed rank test. Exact *p* values shown in **(A)**. ns, non-significant.

To demonstrate the reproducibility of our workflow, this panel was similarly optimized and validated on the Sony ID7000 Spectral Cell Analyzer (**Supplementary Figures 11**-**14**). We did not observe differences in lymphocyte frequencies when compared to the data acquired from the Cytek Aurora flow cytometer (**Figure 5A**), although we did find small discrepancies in marker expression levels, particularly CD25 expression on ILC2s (**Figure 3**; **Supplementary Figures 13**-**14**). Upon clustering and data integration (**Figure 5B**), while lower resolution of the CD4^+^ T cell subset was observed on the Sony instrument, no major differences were noted between flow cytometric data generated using the Cytek and Sony flow cytometers (**Figures 5C, D**). Together, these observations support the implementation and use of this bioinformatics pipeline for reproducibility and accuracy of ILCs in tissues such as tumors.

## Discussion

The identification and characterization of various immune cell subsets within and across tissues impose the use of complex flow cytometric staining panels for accurate analysis of their frequency, numbers, and phenotype. This study reports the design, optimization, and validation of a 25-parameter spectral flow cytometric staining panel that enables the identification of type 1, 2 and 3 ILC subsets in lungs and mammary tumor samples, in addition to various adaptive lymphoid populations. Due to the rarity of ILCs in tissues, a common approach to identify them is assigning one fluorophore with lineage-defining markers to first exclude adaptive lymphocyte populations (6, 30, 31). Studying ILCs in cancer poses a unique challenge, however, as it becomes increasingly clear that immune cells in the tumor microenvironment form a complex network, and neither innate nor adaptive subsets function in isolation. By ensuring that our panel did not group and exclude adaptive lymphocytes, we are able to ascertain similarities and differences of ILC phenotypes to their adaptive counterparts. We acknowledge the absence of antibodies for cytokine detection in our panel, directly limiting our capacity to assess T cell and ILC effector function across tissues and disease conditions. We deliberately separated cytokines from other surface and intracellular markers included in this immunophenotyping panel since the use of *ex vivo* stimulation protocols to assess cytokine expression in lymphocytes can be associated with increased cell death leading to an underestimation of immune cell populations, or potentially change the expression of certain surface markers. As such, the frequency and enumeration of lymphocytes after *ex vivo* stimulation, such as after PMA and Ionomycin stimulation in the presence of Golgi Stop and Golgi Plug, might be inaccurate. Thus, while our panel does not allow for the assessment of immune cell function by the means of cytokine expression analysis, it enables accurate identification of the frequency, number, and phenotype of innate and adaptive lymphocytes in both lung and mammary cancer samples.

The ability to identify the full emission spectrum of a given fluorophore can greatly improve the resolution of a flow cytometry staining panel. This technology also allows for autofluorescence extraction, where researchers can separate the signature of a given tissue’s natural fluorescence and eliminate its influence toward unmixing quality (36, 37). Several autofluorescent signatures can be simultaneously extracted, particularly beneficial when analyzing the heterogeneous environments within tumors. In addition to increased resolution, the large number of parameters that can be accurately unmixed results in high-dimensional sample data. Computational workflows can then be applied to complement the traditional analysis methods of gating strategy and potentially mitigate its limitations. Although reproducible, we observed variation in expression levels of certain markers between paired samples acquired on the Cytek and Sony platforms, analyzed by gating strategy. For example, we observed a larger proportion of CD25^+^ ILC2s in the lungs from data on the Cytek and improved CD4^+^ T cell resolution with the identification of proliferative (Ki67^+^) cells. Unbiased bioinformatics approaches to normalize and integrate data from independent experiments may facilitate more accurate quantification and comparisons in marker expression. It is also important to note that the antibody-conjugate pairs may differ in staining based on cytometer configuration, and considerations must be taken in re-arranging certain parameters in addition to performing instrument-specific antibody titration analyses.

For automated cell clustering of flow cytometric data, we developed and optimized a custom-made bioinformatics pipeline using established R packages, enabling its rapid and easy implementation for users with low coding background performing unbiased flow cytometric analysis. After data cleaning and importation into R, the dataset was further transformed, quality-controlled, normalized, scaled, and analyzed using PCA, UMAP, and unsupervised clustering. This approach enables the comparison of different tissue types, such as tumor and lung samples, eliminating potential variance between conditions. Data transformation is one of the most critical steps; thus, it is essential to test various cofactors, often ranging from hundreds to thousands. However, the appropriate number depends on multiple factors, such as antibody concentration, tissue types, and the instrument. The goal is to ensure maximum resolution between positive and negative peaks while maintaining the negative peak at zero (38). Various publicly available packages can reach similar outcomes. For instance, other packages such as *flowAI (*39), *flowClean (*40), and *flowCut (*41), can be used for quality control. The main advantage of *PeacoQC* over other existing packages is that it can uncover abnormalities in all conventional, spectral, and mass flow cytometric datasets, facilitating the detection of any irregularities. In addition, *PeacoQC* requires relatively less computer memory compared to other described pipelines, facilitating its day-to-day implementation. We implemented the *Seurat* package to perform dimensionality reductions and unsupervised clustering. *Seurat* is one of the most commonly used packages originally designed for single-cell RNA sequencing (scRNAseq), which can be potentially applied to other omics single-cell datasets, including spectral flow cytometry (26). Of note, the number of markers in our flow cytometric panel is significantly lower than the features usually used in scRNAseq; therefore, we recommend decreasing the resolution from the default setting (default *resolution = 1, our pipeline used 0.8*) in the unsupervised *FindCluster* function to avoid over-clustering. Nonetheless, we were unable to identify the ILC3 population using this clustering approach. This might be due to the low percentage of ILC3s found in the tumor and lung, as well as the limitation of computational power; we performed a substantial downsampling before applying dimension reduction and clustering. Including more cells from samples may result in improved cell clustering and refined immune cell subset identification.

Altogether, the implementation of this flow cytometric staining panel or similarly complex antibody panels associated with automated cell clustering will certainly improve the characterization of lymphoid cells in tumors, enabling direct comparisons of marker expression between T cells and ILCs. The robustness of this approach directly pleads for the development, testing, and implementation of similar workflows for the characterization of myeloid cell diversity and the identification of various T cell subsets across tissues, including tumors.

## Code availability

The R scripts and associated FCS files are available at https://github.com/JacquelotLab.

## Data Availability

The data that support the findings of this study are available from the corresponding author upon reasonable request.

## Supplementary material

Supplementary Figure 1 **(A)** Normalized spectral flow cytometric signatures of fluorochromes and unstained tissue autofluorescence across the 64 detectors of a Cytek Aurora. **(B)** Comparison of GATA3 staining resolution between a BV711 conjugated antibody (left) and a PE-Cy7 conjugated antibody (right). **(C)** Representation of co-expressed markers CD3 and CD4 on live CD45^+^ cells in lung samples stained with similar (left) and dissimilar (right) conjugates.

Supplementary Figure 2 Titrations of each antibody on the Cytek Aurora with the selected dilution depicted in red. Antibodies were titrated on spleen, lung, intestinal tissue, and tumor single-cell suspensions.

Supplementary Figure 3 **(A)** Representative cleanup of unmixed lung sample data. **(B)** Representative gating strategy used to identify the major lymphocyte subsets in the lung. NK cells were identified as CD3^-^TCRβ^-^CD19^-^NK1.1^+^NKp46^+^EOMES^+^CD49a^-^; immature NK cell subset was identified as KLRG1^-^CD11b^-^NK cells; M1 NK cell subset was identified as KLRG1^-^CD11b^+^NK cells; M2 NK cell subset was identified as KLRG1^+^CD11b^+^NK cells; ILC1s were identified as CD3^-^TCRβ^-^CD19^-^NK1.1^+^NKp46^+^CD49a^+^EOMES^-^; ILC2s were identified as CD3^-^TCRβ^-^CD19^-^NK1.1^-^NKp46^-^CD4^-^CD8^-^CD90.2^+^CD127^+^GATA3^+^RORγt^-^; ILC3s were identified as CD3^-^TCRβ^-^CD19^-^NK1.1^-^NKp46^-^CD4^-^CD8^-^CD90.2^+^CD127^+^RORγt^+^. **(C)** Proportion of each cell subset as a percentage of total live CD45^+^ leukocytes (left) and total cell counts of subsets in the lung (right). **(D)** Proportion of NK cell subsets as a percentage of total NK cells (left) and total cell count in the lung (right). Bar graphs show the mean ± SD of 3 biological replicates.

Supplementary Figure 4 **(A)** Representative histograms of marker expression on T and B cells in the lung and tumor annotated with mean expression ± SD. **(B)** Heatmap representing mean surface marker expression of T and B cells in the lung (top) and tumor (bottom). n=3 lung, n=4 tumor.

Supplementary Figure 5 **(A-B)** Histograms of each marker’s fluorescence in the lung and tumor samples before and after transformation or normalization.

Supplementary Figure 6 **(A-B)** Heatmaps of protein expression level in each cell after unsupervised clustering with **(A)** or without integration **(B)**.

Supplementary Figure 7 Feature plots showing the expression level of each protein in the lungs before integration.

Supplementary Figure 8 Feature plots showing the expression level of each protein in the tumors before integration.

Supplementary Figure 9 Feature plots showing the expression level of each protein in the lungs after integration.

Supplementary Figure 10 Feature plots showing the expression level of each protein in the tumors after integration.

Supplementary Figure 11 Titrations of each antibody on the Sony ID7000 with the selected dilution depicted in red. Antibodies were titrated on spleen, lung, intestinal tissue, and tumor single-cell suspensions.

Supplementary Figure 12 **(A)** Representative cleanup of unmixed lung sample data. Representative gating strategy used to identify the major lymphocyte subsets in the lung **(B)** and tumor **(C)**. NK cells were identified as CD3^-^TCRβ^-^CD19^-^NK1.1^+^NKp46^+^EOMES^+^CD49a^-^; immature NK cell subset was identified as KLRG1^-^CD11b^-^NK cells; M1 NK cell subset was identified as KLRG1^-^CD11b^+^NK cells; M2 NK cell subset was identified as KLRG1^+^CD11b^+^NK cells; ILC1s were identified as CD3^-^TCRβ^-^CD19^-^NK1.1^+^NKp46^+^CD49a^+^EOMES^-^; ILC2s were identified as CD3^-^TCRβ^-^CD19^-^NK1.1^-^NKp46^-^CD4^-^CD8^-^CD90.2^+^CD127^+^GATA3^+^RORγt^-^; ILC3s were identified as CD3^-^TCRβ^-^CD19^-^NK1.1^-^NKp46^-^CD4^-^CD8^-^CD90.2^+^CD127^+^RORγt^+^. **(D)** Proportion of subsets as a percentage of total CD45^+^ leukocytes and cell counts of lung (left) and tumor (right) subsets. Tumor cell counts calculated as cells per gram of tumor tissue. Bar graphs show the mean ± SD. n=3 lung, n=4 tumor. Data acquired on the Sony ID7000.

Supplementary Figure 13 **(A)** Representative histograms of marker expression on ILC subsets in the lung and tumor annotated with mean expression ± SD. **(B**) Heatmap representing mean surface marker expression of ILCs in the lung (top) and tumor (bottom). n=3 lung, n=4 tumor. Data acquired on the Sony ID7000.

Supplementary Figure 14 **(A)** Representative histograms of marker expression on T and B cells in the lung and tumor annotated with mean expression ± SD. **(B)** Heatmap representing mean surface marker expression of T and B cells in the lung (top) and tumor (bottom). n=3 lung, n=4 tumor. Data acquired on the Sony ID7000.
