# Supplementary figures and images for: Development and computational analysis of high dimensional spectral flow cytometry data for the resolution of innate lymphoid cells in the mammary tumor microenvironment
Source: Front Immunol. 2026 Jan 27;17:1730567. doi: 10.3389/fimmu.2026.1730567 (PMC12886383; doi:10.3389/fimmu.2026.1730567)

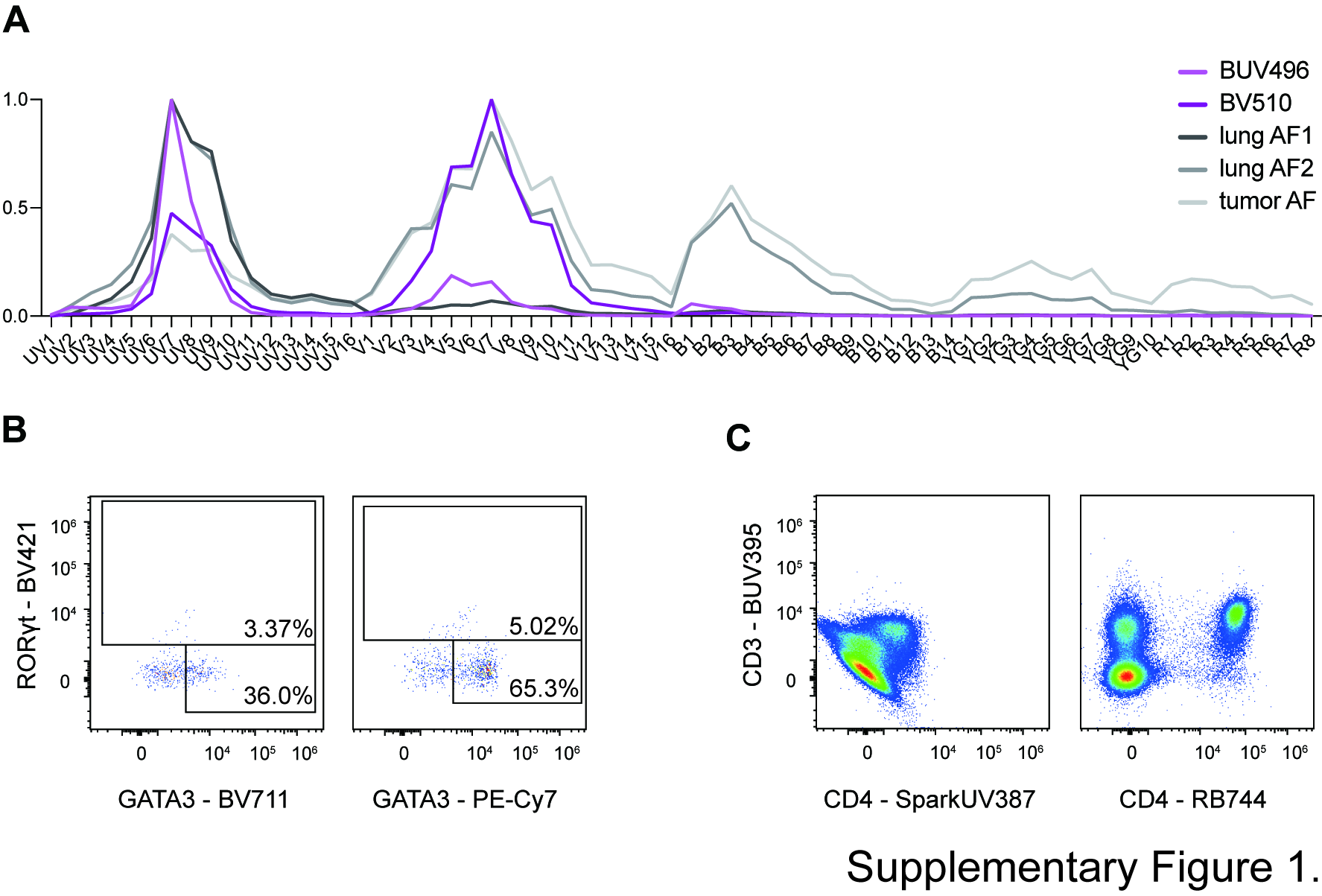

Supplement: Supplementary Figure 1 — (A) Normalized spectral flow cytometric signatures of fluorochromes and unstained tissue autofluorescence across the 64 detectors of a Cytek Aurora. (B) Comparison of GATA3 staining resolution between a BV711 conjugated antibody (left) and a PE-Cy7 conjugated antibody (right). (C) Representation of co-expressed markers CD3 and CD4 on live CD45+ cells in lung samples stained with similar (left) and dissimilar (right) conjugates. [file Image1.tif]

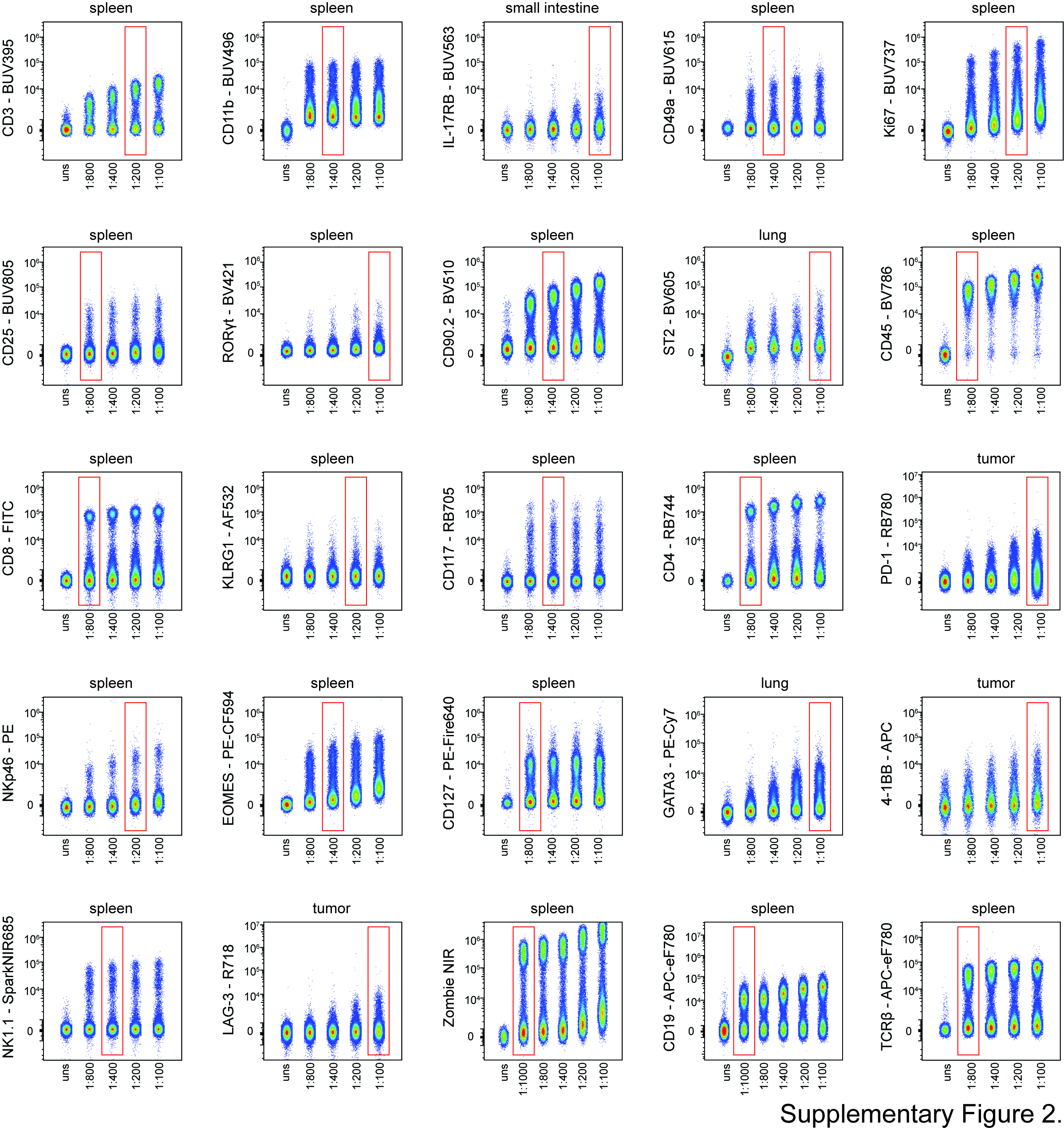

Supplement: Supplementary Figure 2 — Titrations of each antibody on the Cytek Aurora with the selected dilution depicted in red. Antibodies were titrated on spleen, lung, intestinal tissue, and tumor single-cell suspensions. [file Image2.tif]

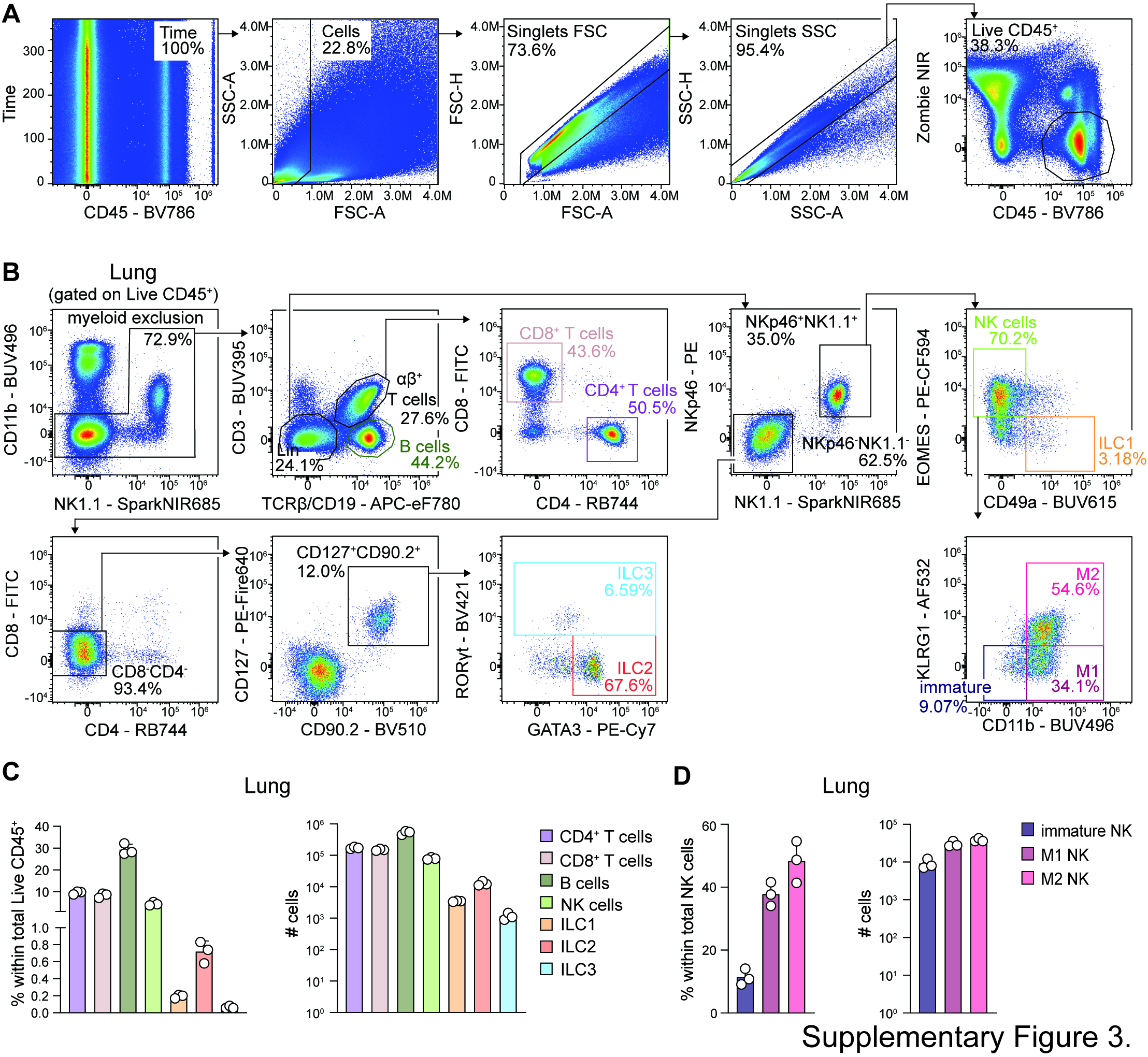

Supplement: Supplementary Figure 3 — (A) Representative cleanup of unmixed lung sample data. (B) Representative gating strategy used to identify the major lymphocyte subsets in the lung. NK cells were identified as CD3-TCRβ-CD19-NK1.1+NKp46+EOMES+CD49a-; immature NK cell subset was identified as KLRG1-CD11b-NK cells; M1 NK cell subset was identified as KLRG1-CD11b+NK cells; M2 NK cell subset was identified as KLRG1+CD11b+NK cells; ILC1s were identified as CD3-TCRβ-CD19-NK1.1+NKp46+CD49a+EOMES-; ILC2s were identified as CD3-TCRβ-CD19-NK1.1-NKp46-CD4-CD8-CD90.2+CD127+GATA3+RORγt-; ILC3s were identified as CD3-TCRβ-CD19-NK1.1-NKp46-CD4-CD8-CD90.2+CD127+RORγt+. (C) Proportion of each cell subset as a percentage of total live CD45+ leukocytes (left) and total cell counts of subsets in the lung (right). (D) Proportion of NK cell subsets as a percentage of total NK cells (left) and total cell count in the lung (right). Bar graphs show the mean ± SD of 3 biological replicates. [file Image3.tif]

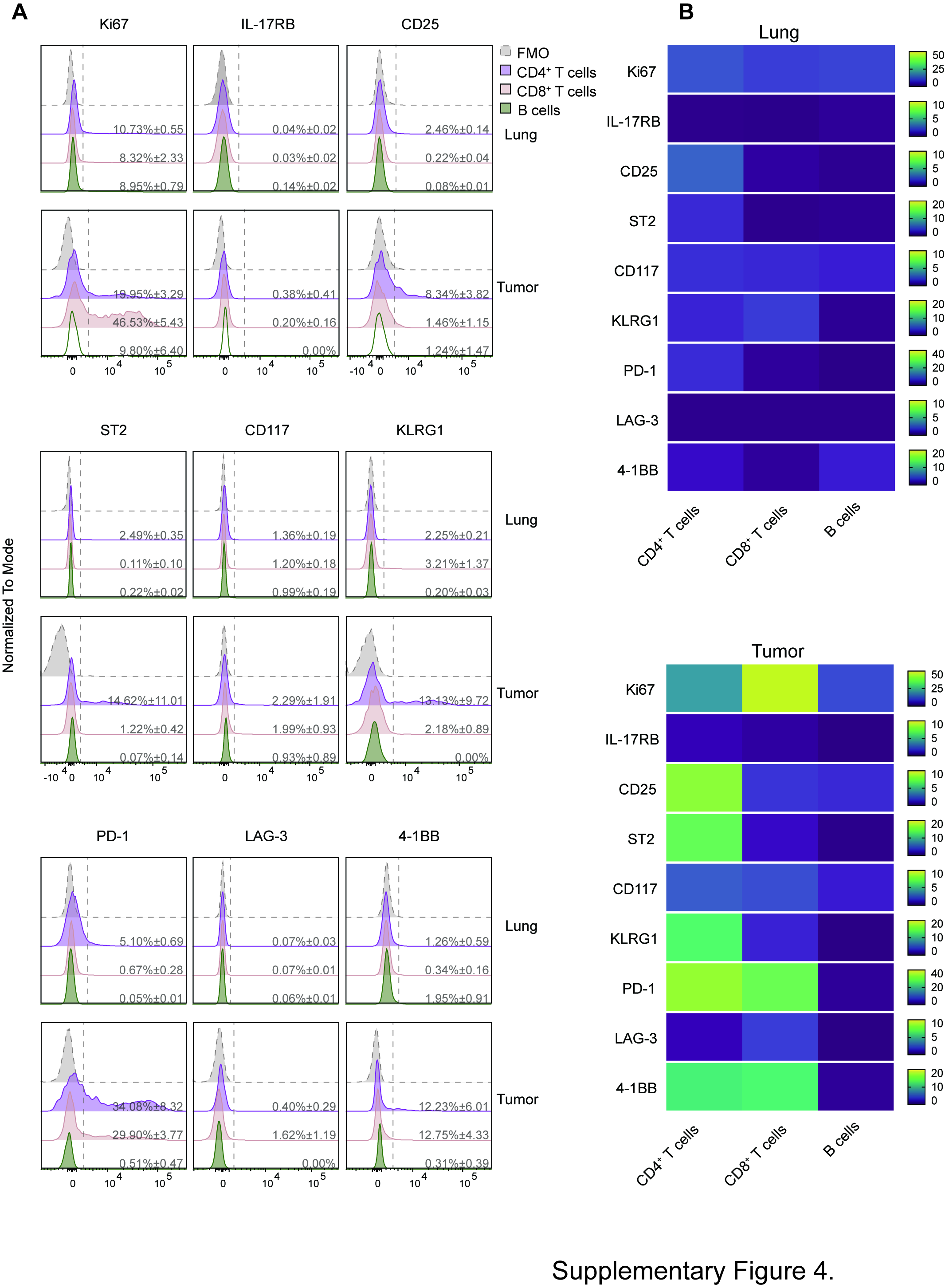

Supplement: Supplementary Figure 4 — (A) Representative histograms of marker expression on T and B cells in the lung and tumor annotated with mean expression ± SD. (B) Heatmap representing mean surface marker expression of T and B cells in the lung (top) and tumor (bottom). n=3 lung, n=4 tumor. [file Image4.tif]

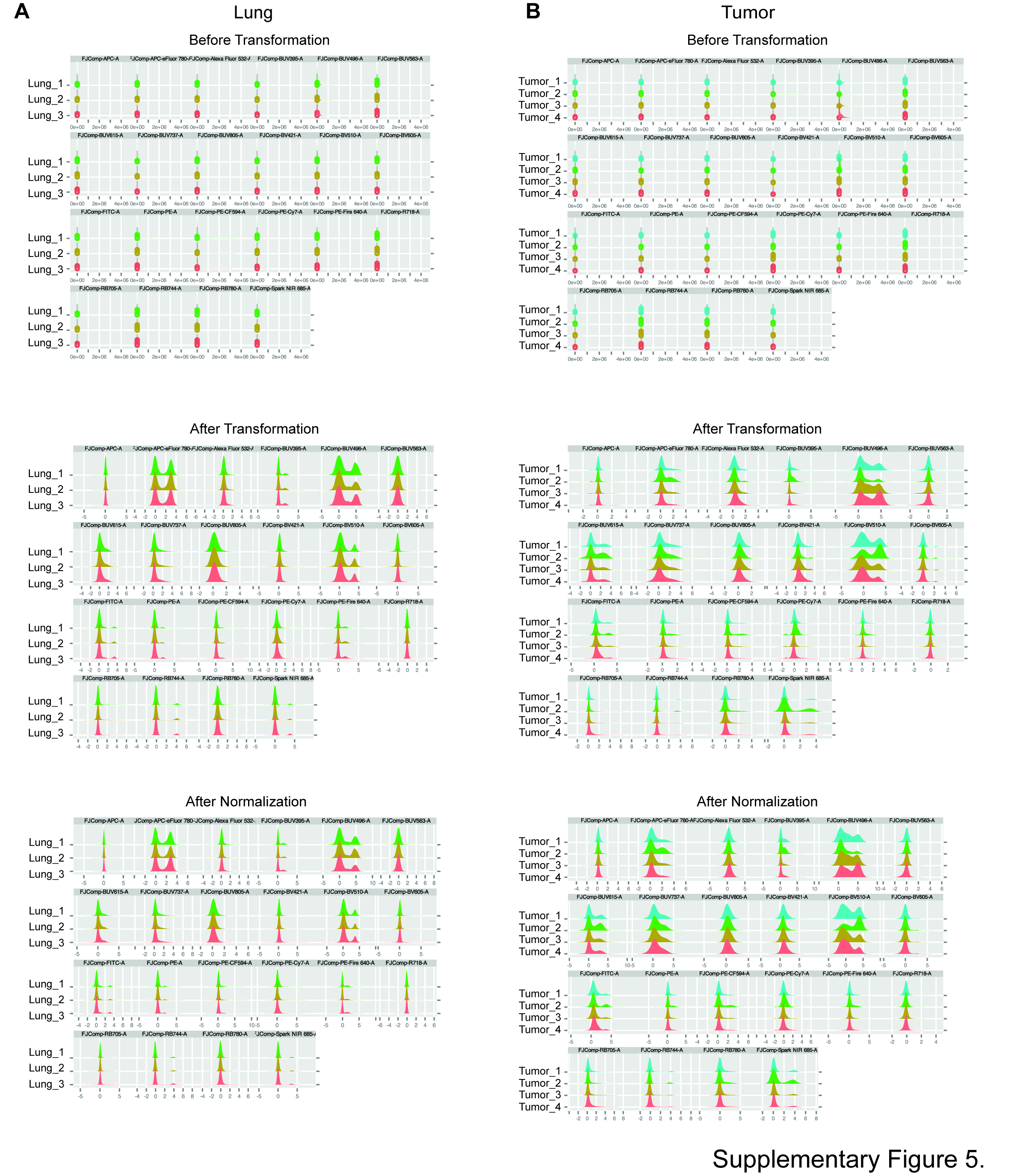

Supplement: Supplementary Figure 5 — (A-B) Histograms of each marker’s fluorescence in the lung and tumor samples before and after transformation or normalization. [file Image5.tif]

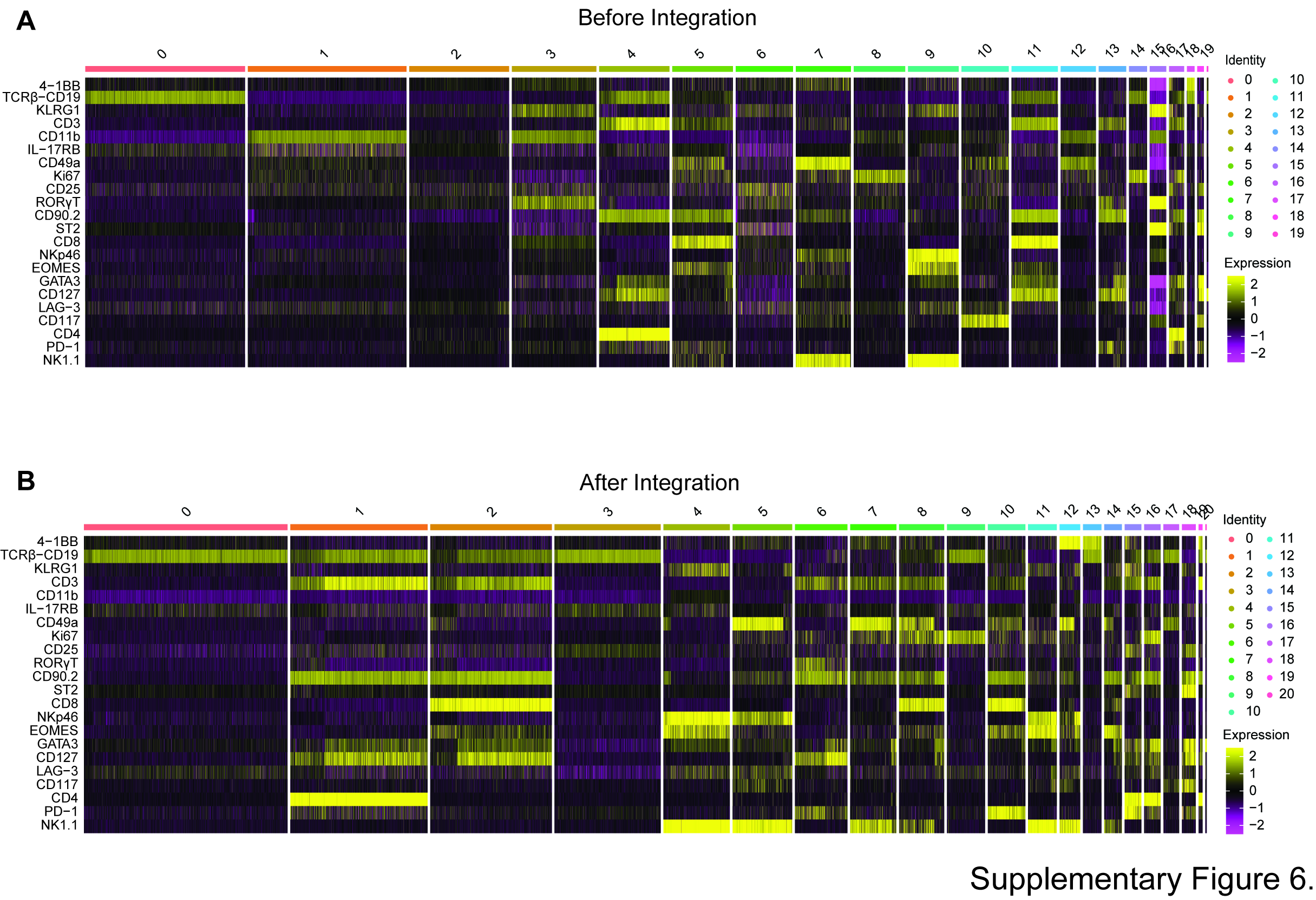

Supplement: Supplementary Figure 6 — (A-B) Heatmaps of protein expression level in each cell after unsupervised clustering with (A) or without integration (B). [file Image6.tif]

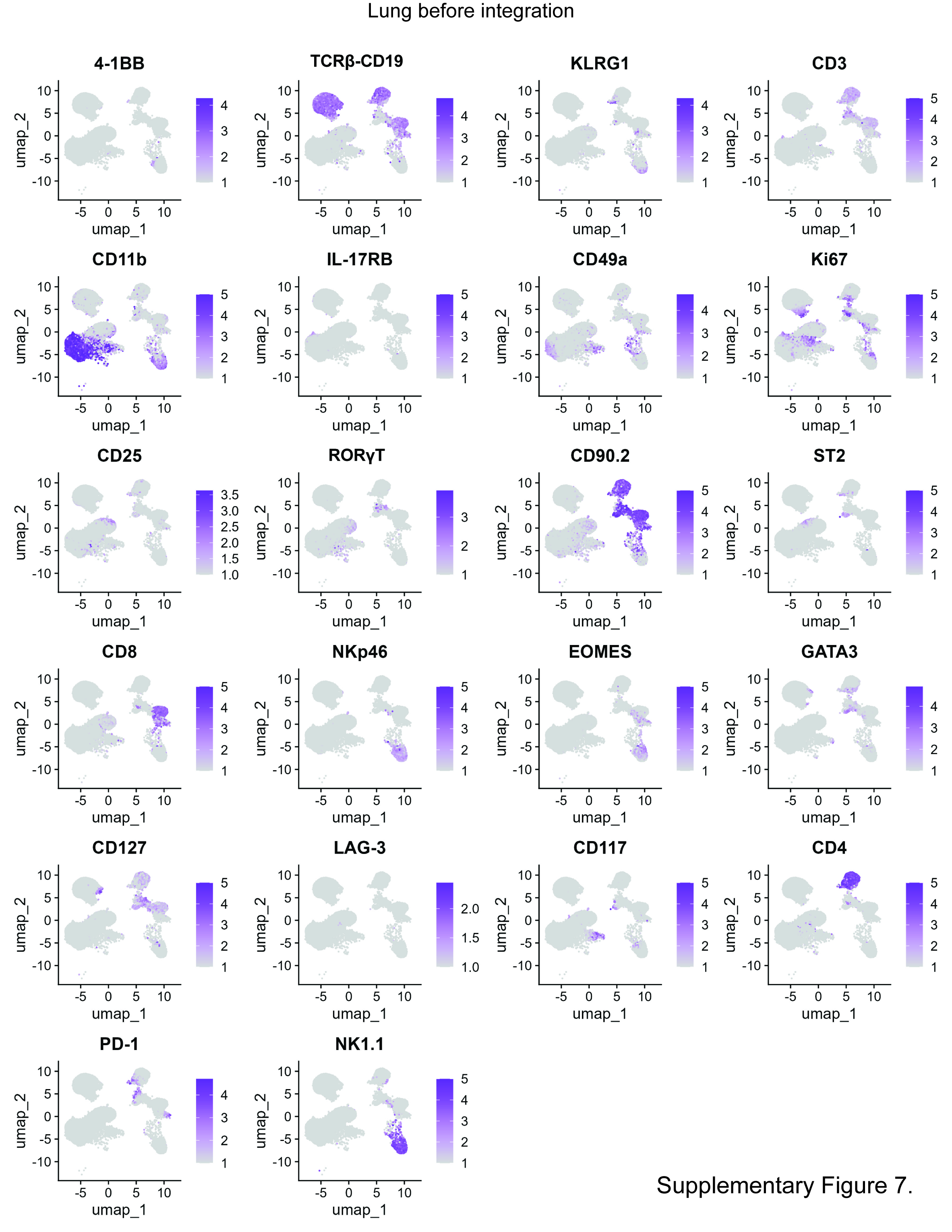

Supplement: Supplementary Figure 7 — Feature plots showing the expression level of each protein in the lungs before integration. [file Image7.tif]

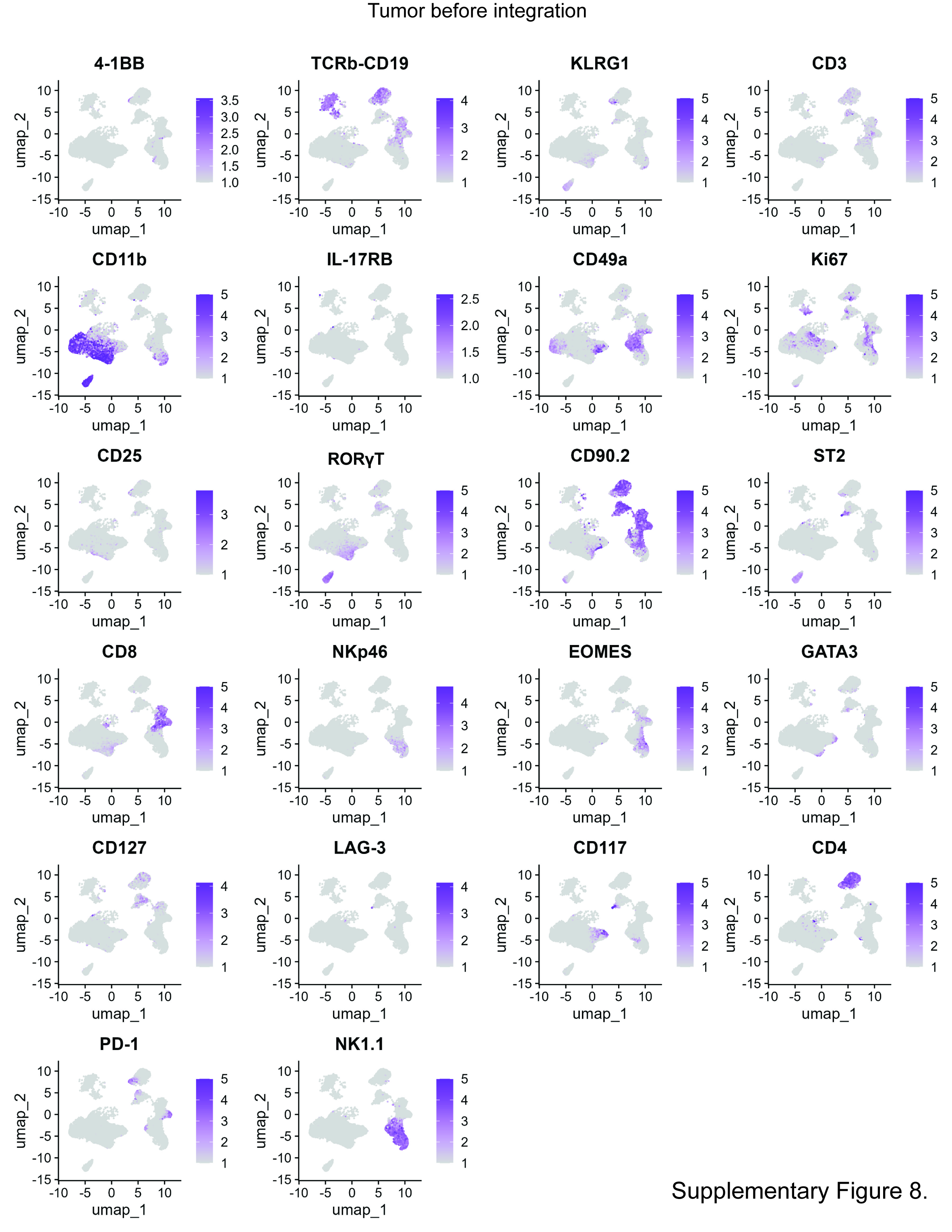

Supplement: Supplementary Figure 8 — Feature plots showing the expression level of each protein in the tumors before integration. [file Image8.tif]

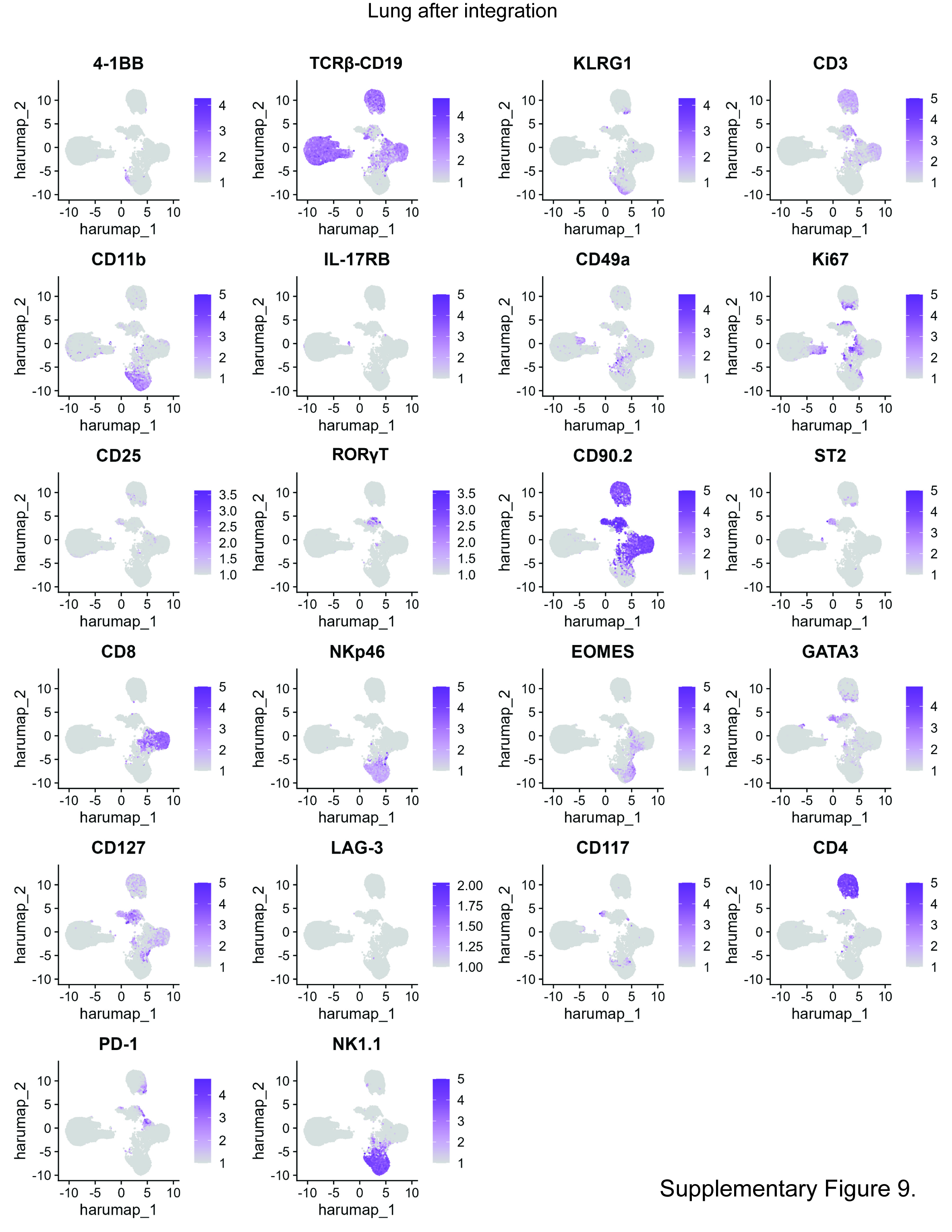

Supplement: Supplementary Figure 9 — Feature plots showing the expression level of each protein in the lungs after integration. [file Image9.tif]

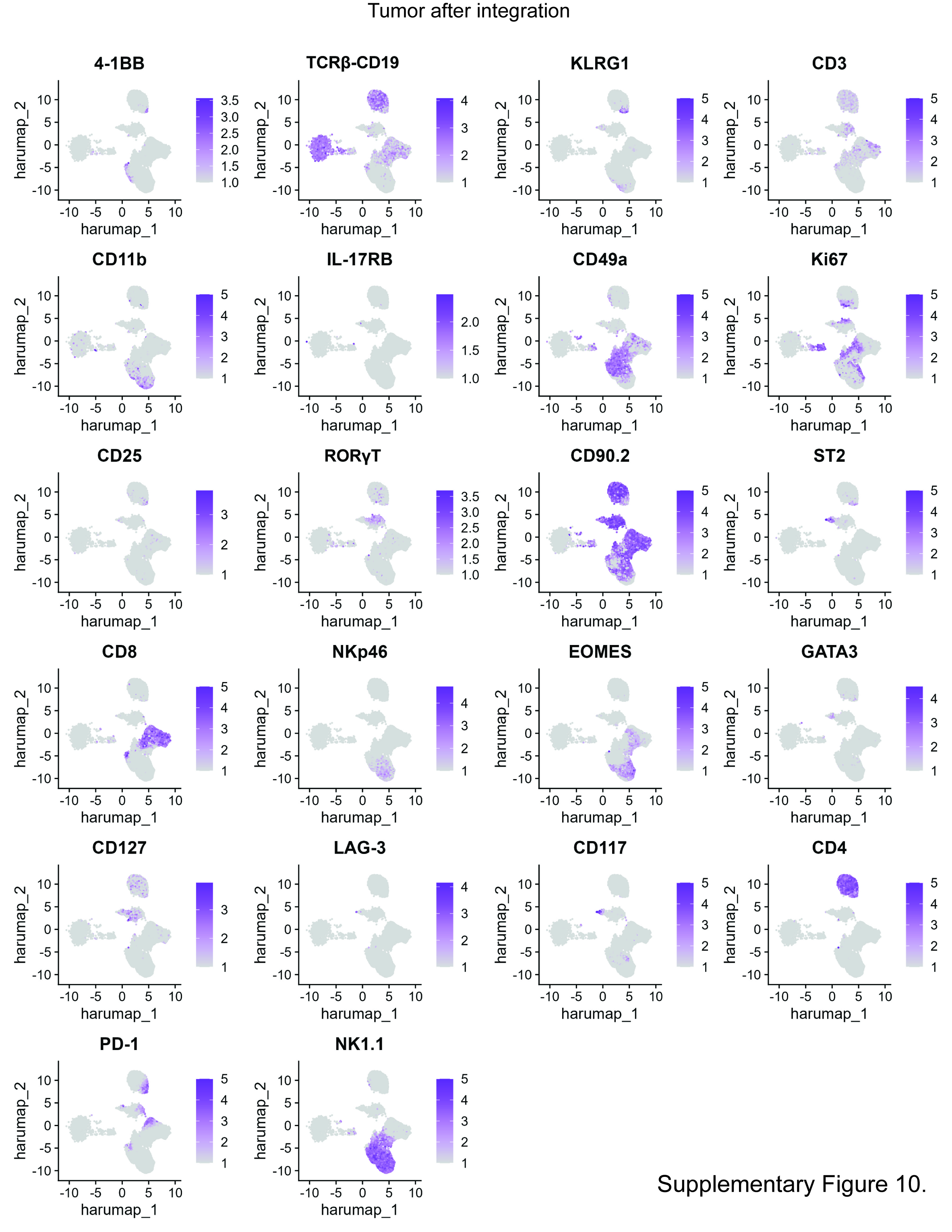

Supplement: Supplementary Figure 10 — Feature plots showing the expression level of each protein in the tumors after integration. [file Image10.tif]

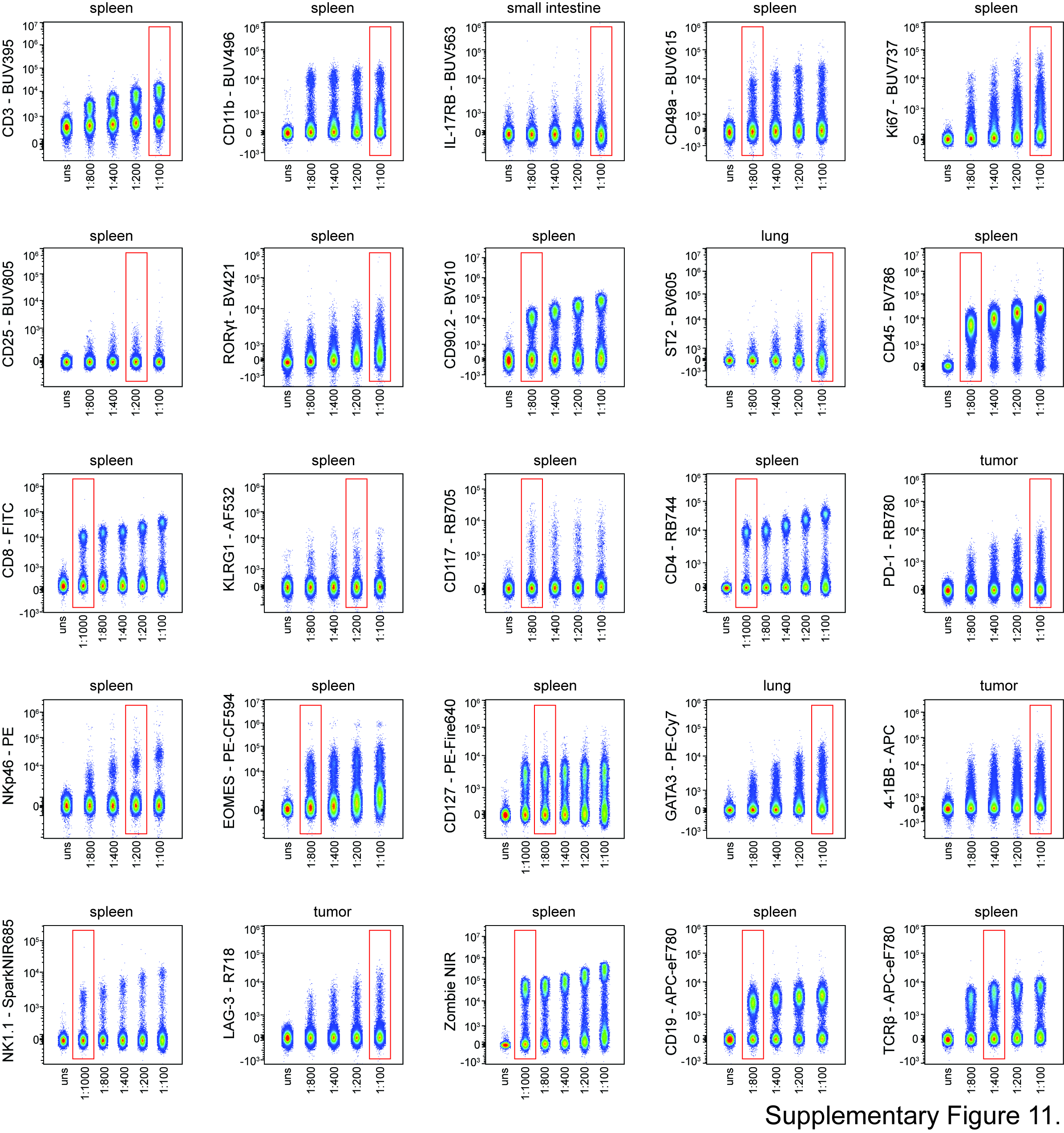

Supplement: Supplementary Figure 11 — Titrations of each antibody on the Sony ID7000 with the selected dilution depicted in red. Antibodies were titrated on spleen, lung, intestinal tissue, and tumor single-cell suspensions. [file Image11.tif]

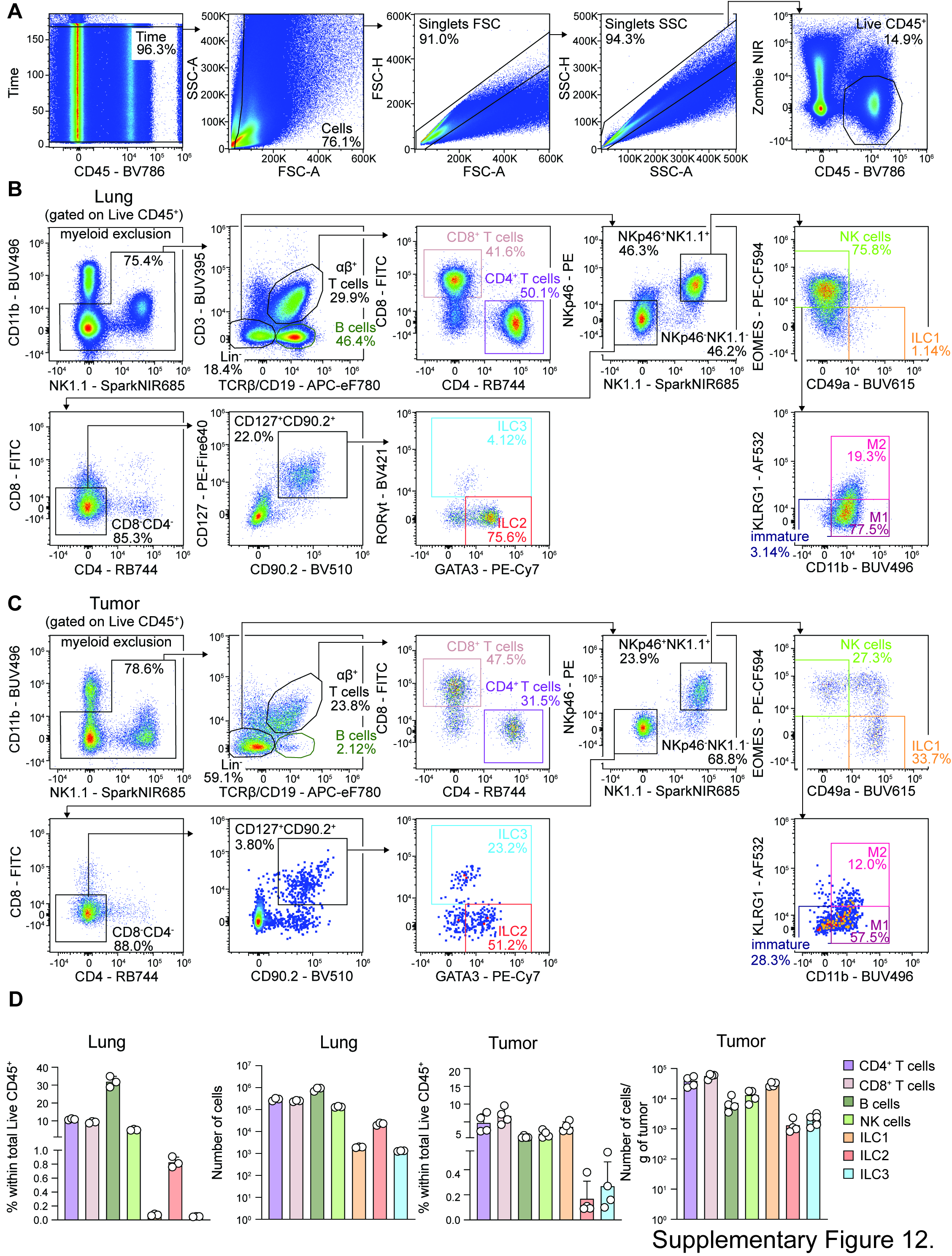

Supplement: Supplementary Figure 12 — (A) Representative cleanup of unmixed lung sample data. Representative gating strategy used to identify the major lymphocyte subsets in the lung (B) and tumor (C). NK cells were identified as CD3-TCRβ-CD19-NK1.1+NKp46+EOMES+CD49a-; immature NK cell subset was identified as KLRG1-CD11b-NK cells; M1 NK cell subset was identified as KLRG1-CD11b+NK cells; M2 NK cell subset was identified as KLRG1+CD11b+NK cells; ILC1s were identified as CD3-TCRβ-CD19-NK1.1+NKp46+CD49a+EOMES-; ILC2s were identified as CD3-TCRβ-CD19-NK1.1-NKp46-CD4-CD8-CD90.2+CD127+GATA3+RORγt-; ILC3s were identified as CD3-TCRβ-CD19-NK1.1-NKp46-CD4-CD8-CD90.2+CD127+RORγt+. (D) Proportion of subsets as a percentage of total CD45+ leukocytes and cell counts of lung (left) and tumor (right) subsets. Tumor cell counts calculated as cells per gram of tumor tissue. Bar graphs show the mean ± SD. n=3 lung, n=4 tumor. Data acquired on the Sony ID7000. [file Image12.tif]

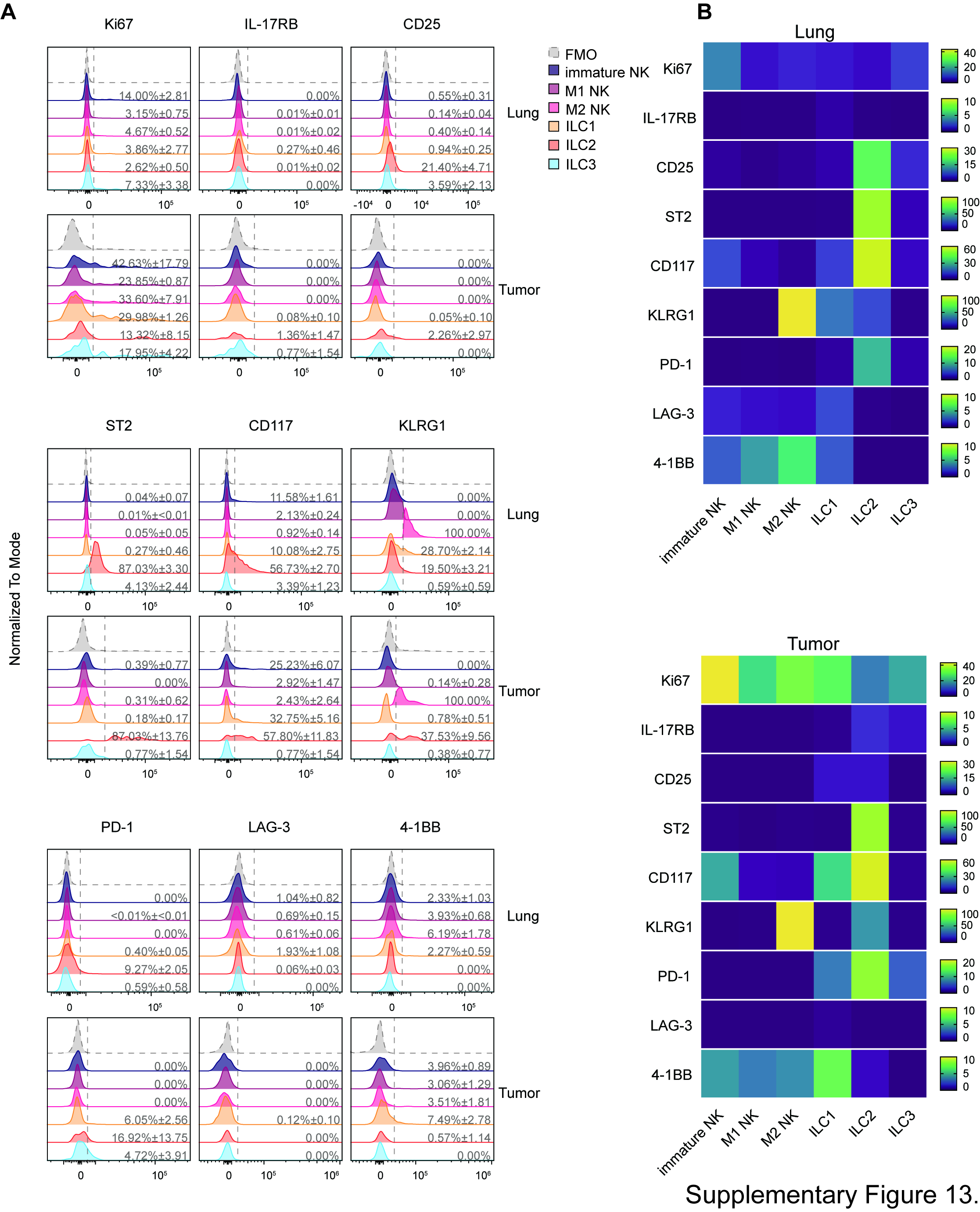

Supplement: Supplementary Figure 13 — (A) Representative histograms of marker expression on ILC subsets in the lung and tumor annotated with mean expression ± SD. (B) Heatmap representing mean surface marker expression of ILCs in the lung (top) and tumor (bottom). n=3 lung, n=4 tumor. Data acquired on the Sony ID7000. [file Image13.tif]

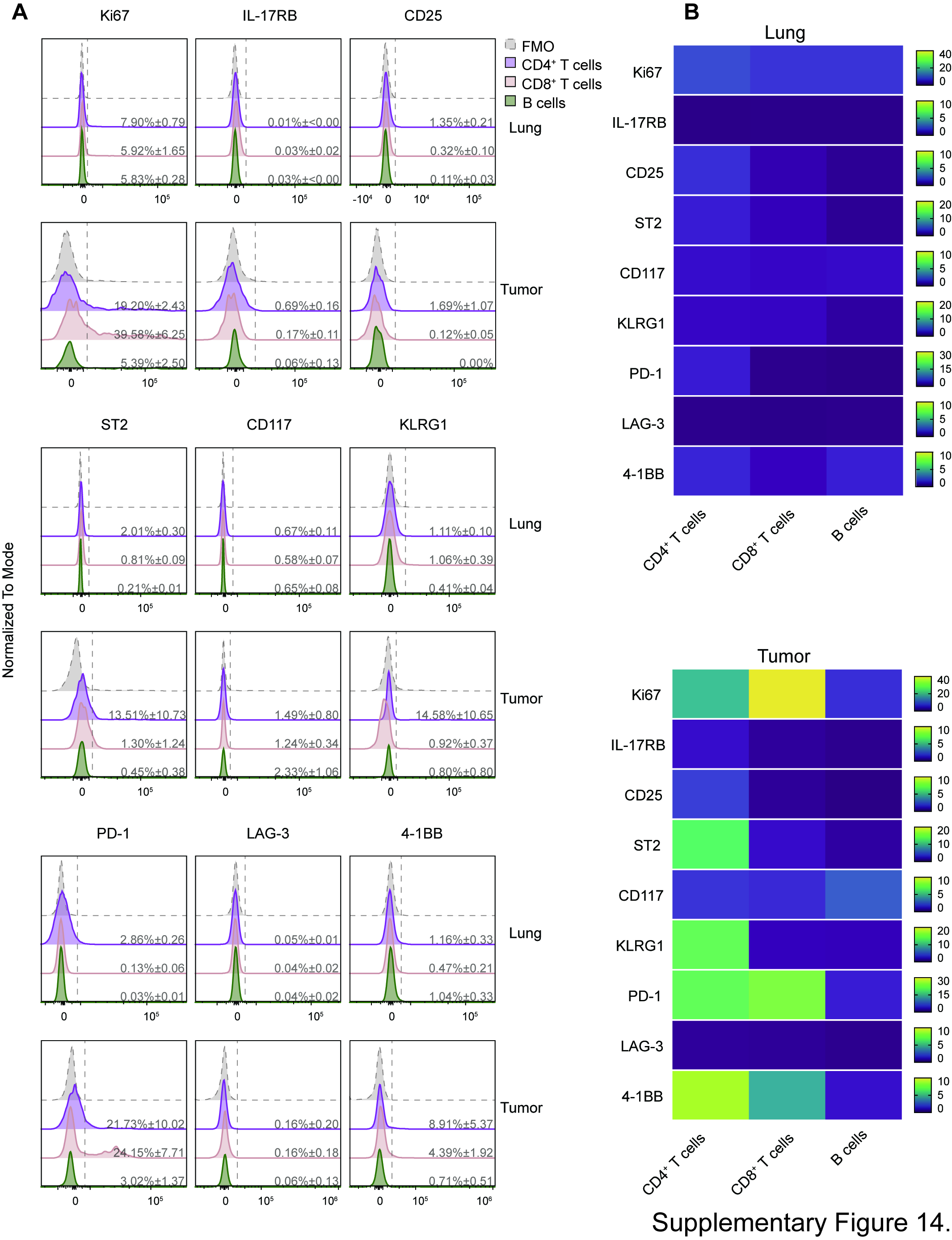

Supplement: Supplementary Figure 14 — (A) Representative histograms of marker expression on T and B cells in the lung and tumor annotated with mean expression ± SD. (B) Heatmap representing mean surface marker expression of T and B cells in the lung (top) and tumor (bottom). n=3 lung, n=4 tumor. Data acquired on the Sony ID7000. [file Image14.tif]
